# Cellular Distribution and Motion of Essential Magnetosome Proteins Expressed in Mammalian Cells

**DOI:** 10.3390/bios15120797

**Published:** 2025-12-04

**Authors:** Qin Sun, Cécile Fradin, Moeiz Ahmed, R. Terry Thompson, Frank S. Prato, Donna E. Goldhawk

**Affiliations:** 1 Imaging, Lawson Research Institute, London, ON N6A 4V2, Canada; qsun@lawsonimaging.ca (Q.S.); mahmed2027@meds.uwo.ca (M.A.); thompson@lawsonimaging.ca (R.T.T.); prato@lawsonimaging.ca (F.S.P.); 2 Medical Biophysics, Western University, London, ON N6A 3K7, Canada; 3 Collaborative Graduate Program in Molecular Imaging, Western University, London, ON N6A 3K7, Canada; 4 Physics & Astronomy, McMaster University, Hamilton, ON L8S 4L8, Canada; fradin@mcmaster.ca; 5 Biochemistry & Biomedical Sciences, McMaster University, Hamilton, ON L8S 4L8, Canada; 6 Medical Imaging, Western University, London, ON N6A 3K7, Canada

**Keywords:** molecular imaging, microparticles, biosensor, nanotechnology, magnetosome, mobility, mammalian cells

## Abstract

Magnetosomes are organelle-like structures within magnetotactic bacteria that store iron biominerals in membrane-bound vesicles. In bacteria, formation of these structures is highly regulated by approximately 30 genes, which are conserved throughout different species. To compartmentalize iron in mammalian cells and provide gene-based contrast for magnetic resonance imaging, we introduced key magnetosome proteins. The expression of essential magnetosome genes *mamI* and *mamL* as fluorescent fusion proteins in a human melanoma cell line confirmed their co-localization and interaction. Here, we investigate the expression of two more essential magnetosome genes, *mamB* and *mamE*, using confocal microscopy to describe fluorescent fusion protein expression patterns and analyze the observed intracellular mobility. Custom software was developed to characterize fluorescent particle trajectories. In mammalian cells, essential magnetosome proteins display different diffusive behaviours. However, all magnetosome proteins travelled at similar velocities when interacting with mammalian mobile elements, suggesting that MamL, MamL + MamI, MamB, and MamE interact with similar molecular motor proteins. These results confirm that localization and interaction of essential magnetosome proteins are feasible within the mammalian intracellular compartment.

## 1. Introduction

In magnetotactic bacteria (MTB), magnetosome biosynthesis allows the cell to compartmentalize iron biominerals in membrane-enclosed vesicles [1,2]. In this way, MTB may respond to external magnetic fields: principally the geomagnetic field (µTesla range) exerted on MTB in their natural habitat [3]. Transferring of this magnetic functionality to a variety of cell types could enable medical imaging applications, including magnetic resonance imaging (MRI; Tesla range) and the possibility of tracking cells using gene-based contrast [4] and reporter gene expression [5]. Molecular imaging with MRI has demonstrated clinical utility; however, it relies heavily on exogenous superparamagnetic iron oxide (SPIO) nanoparticles that cannot report cellular and molecular activities throughout the cell’s life cycle. To improve molecular MRI, the magnetosome offers a protein-directed solution, which may be compatible with multiple cell types.

Magnetosome biosynthesis is a stepwise process that begins with vesicle formation and culminates with iron biomineralization. This entire process is regulated by approximately 30 genes, the majority of which are located on the magnetosome genomic island [6,7]. Many of these genes encode membrane proteins, which are involved in different steps of bacterial magnetosome formation: inner membrane invagination leading to vesicle formation, recruitment of proteins to the vesicle membrane, alignment of vesicles along a protein filament, concentration of iron inside the vesicle and finally magnetic crystal nucleation and growth. To stimulate the formation of a rudimentary magnetosome-like particle in mammalian cells, we introduced select genes (alone or in combination) deemed essential for the initial stages of magnetosome formation, namely *mamI*, *mamL*, *mamB* and *mamE*. These four genes are clustered on the *mamAB* operon and are highly conserved in most species of MTB [8]. The gene products of *mamI*, *mamL*, and *mamB* are believed to play an essential role [9] in the first steps of magnetosome vesicle formation [6,10], including designation of the vesicle and recruitment of other magnetosome-associated proteins like MamE [11,12]. The latter plays a crucial role in the initiation of iron biomineralization and recruitment of additional magnetosome-associated proteins [13].

To examine the structure and function of magnetosome components in prokaryotes, a wide variety of studies have used fluorescent proteins fused to magnetosome proteins, including GFP constructs of MamC, MamF, MamG [14] and MamI [15] and mNeonGreen constructs of MamB, MamQ and MamK [16]. To better understand the roles of MamI, MamL, MamB and MamE in a mammalian cell type [17], we expressed these essential magnetosome proteins with fluorescent tags and evaluated their ability to (co)localize and interact in the intracellular membranous compartment. Analysis of transgene function indicates that each of these magnetosome proteins retains iron-handling characteristics that influence MRI relaxation rates [18]. In addition, confocal microscopy and a red fluorescent MamL fusion protein show that Tomato-MamL assembles into punctate structures that move intracellularly in a mammalian cell line [19]. These punctate structures efficiently recruited the green fluorescent MamI fusion protein, EGFP-MamI, through magnetosome protein–protein interactions. In contrast, EGFP-MamI expressed alone in mammalian cells had a very different cellular distribution [19]. The novel and unexpected pattern of mobility associated with the particles formed by these magnetosome proteins, or the particles to which they attach in a foreign cell environment, prompted further investigation into particle trajectory analysis.

Here, we quantitatively compare the distribution and mobility of a larger set of magnetosome proteins, expressed as fluorescent fusion proteins in a mammalian cell line (Tomato-MamL, EGFP-MamI, Tomato-MamB, EGFP-MamE). When expressed on their own, Tomato-MamL, Tomato-MamB and EGFP-MamE all form (or localize to) punctate structures, which are mostly mobile in the case of MamL and MamB, but immobile in the case of MamE. For EGFP-MamI, co-expression with MamL was necessary to ensure a similar localization to punctate structures so that a comparison of their mobilities could be examined. These punctate structures will herein be referred to as “particles”. We tracked fluorescent particle motion and described the mean-squared displacement (MSD) with a simple power-law analysis to distinguish between different types of mobility. With these tools, the trajectories of punctate intracellular structures were classified into three different categories corresponding to confined, diffusing and actively transported particles. The motions detected for MamL and MamB (and for MamI when interacting with MamL) show that a portion of these structures display directed motion. This demonstrates the capacity of magnetosome proteins to spontaneously interact with elements of eukaryotic transport machinery.

## 2. Materials and Methods

### 2.1. Molecular Cloning

Magnetosome genes *mamI* and *mamL* were amplified by PCR from the genomic DNA of *Magnetospirillum magneticum* strain AMB-1 (ATCC 700264) using custom primers [19]. Briefly, *mamI* and *mamL* amplicons were purified using a PCR clean-up kit (Invitrogen, Life Technologies, Burlington, ON, Canada); digested with appropriate restriction enzymes; and purified once more, prior to insertion in the cloning vectors pEGFP-C1 (Clontech, Mountain View, CA, USA) and ptdTomato-C1 (Clontech), respectively. All vector-insert constructs used for mammalian cell transfection were propagated in *Escherichia coli* XL10GOLD and verified by sequencing.

Sequence analysis of the MamL protein indicates the presence of a positively-charged peptide [6] in the C-terminal 15 amino acids. Cationic peptides also known as cell-penetrating peptides have been implicated in endocytotic pathways [20]. For cloning Tomato-MamL_trunc_, the last 15 amino acids from the C-terminus of MamL were removed by site-directed mutagenesis [19]. Briefly, primers were designed to include a stop codon 45 nucleotides upstream from the termination of *mamL*. These primers were used in PCR amplification of the *mamL_trunc_* gene, which was then inserted into the ptdTomato-C1 vector with restriction enzymes EcoRI and BglII.

For cloning FLAG-MamL, primers were designed to flank the *mamL* gene and include an N-terminal FLAG sequence for immunodetection [19]. The insert was amplified using PCR, purified using a PCR clean-up kit (Invitrogen, Life Technologies, Burlington, ON, Canada), and restriction digested using SacI and EcoRI. *FLAG-mamL* was then inserted into the pSF-EMCV-*FLuc* vector and propagated in *Escherichia coli* strain XL10GOLD.

For cloning Tomato-MamB, the *mamB* gene was amplified by PCR from AMB-1 genomic DNA using custom primers (Table 1). The amplicon was purified with a PCR clean-up kit, digested with restriction enzymes (Table 1), then purified and inserted into ptdTomato-C1.

For cloning EGFP-MamE, the *mamE* gene was amplified by PCR from the AMB-1 genomic DNA using custom primers (Table 1). The amplicon was purified with a PCR clean-up kit, digested with restriction enzymes (Table 1), then purified and inserted into the pEGFP-C1.

Several attempts at cloning and sequencing EGFP-*mamE* revealed multiple constructs with sporadic mutations relative to the consensus sequence, which itself consists of variable and conserved regions [12] (Supplementary Figure S1). Two of these mutations, denoted EGFP-*mamE* (G49S, T641S) and EGFP-*mamE* (T317A), were used for confocal microscopy and provided comparable results. The sequence of each construct can be accessed in the Electronic Thesis and Dissertation Repository maintained by Scholarship@Western using the following URL: https://hdl.handle.net/20.500.14721/32958 (accessed on 5 April 2023) [21].

### 2.2. Cell Culture

MDA-MB-435 cells (ATCC HTB-129; derived from an adult female and characterized as a melanoma cell line) are a model of aggressive tumorigenesis [22]. Cells were cultured in 100 mm cell culture dishes (CELLSTAR, VWR International, Mississauga, ON, Canada) with Dulbecco’s Modified Eagle Medium (DMEM) containing 1 g/L glucose (Gibco, Life Technologies, Burlington, ON, Canada), 10% fetal bovine serum (FBS; Gibco), 4 U/mL penicillin, and 4 µg/mL streptomycin at 37 °C with 5% CO_2_.

To create cell lines expressing red fluorescent fusion proteins tdTomato (Tomato)-MamL, Tomato-MamL_trunc,_ or Tomato-MamB, the enhanced green fluorescent protein (EGFP) fusion protein EGFP-MamE, or the FLAG (DYKDDDDK) tagged protein FLAG-MamL, cells were grown to 60–70% confluency on a 100 mm dish and transfected using Lipofectamine 2000 (Invitrogen), according to company protocol, using 8 µg of each construct. After 16 h, cells were placed in full medium for 48 h before commencing antibiotic selection. To select stable cell lines, cells were grown in the presence of 500 µg/mL Geneticin (G418; Gibco) for Tomato or EGFP expression systems and 500 ng/mL Puromycin (Gibco) for the FLAG expression system.

For co-expression of both pEGFP-*mamI* and ptdTomato-*mamL,* cells stably expressing Tomato-MamL were transfected with 8 µg of pEGFP-*mamI*. After 16 h, cells were placed in full medium for 48 h before commencing antibiotic selection. Cells were then sorted using fluorescence-activated cell sorting (FACS; London Regional Flow Cytometry Facility, Robarts Research Institute, London, ON, Canada) to obtain a population of cells fluorescing both green and red (i.e., expressing both EGFP-MamI and Tomato-MamL, respectively). For co-expression with the pSF-FLAG-*mamL*-EMCV-*FLuc* construct, MDA-MB-435 cells expressing EGFP-MamI were grown to 60–70% confluency on a 100 mm dish and transfected using Lipofectamine 2000 (Invitrogen), according to company protocol, using 8 µg of pSF-FLAG-*mamL*-EMCV-*FLuc*. After 16 h, cells were placed in full medium for 48 h before commencing antibiotic selection. To select stable cell lines, cells were grown in the presence of 500 µg/mL Geneticin (G418; Gibco) and 500 ng/mL Puromycin (Gibco).

### 2.3. Protein Sample Preparation

Stably transfected cells were cultured to 70% confluency on a 100 mm dish, then washed twice using 10 mL phosphate-buffered saline pH 7.4 (PBS, 137 mM NaCl/2.7 mM KCl/10 mM Na_2_HPO_4_). Four to five dishes of cells were then collected into a 1 mL lysis solution containing 850 μL radioimmunoprecipitation assay buffer (RIPA, 10 mM Tris-HCl pH 7.5/140 mM NaCl/1% NP-40/1% sodium deoxycholate/0.1% sodium dodecyl sulfate (SDS)) and 150 μL of Complete Mini protease inhibitor cocktail (Roche Diagnostic Systems, Laval, QC, Canada). Harvested cells were sonicated using three 12-s bursts of a Sonic Dismembrator (model 500, Thermo Fisher Scientific, Ottawa, ON, Canada) at an amplitude of 30%. Total amount of protein was quantified using the BCA assay [23].

### 2.4. Western Blot

Protein samples of MDA-MB-435 cells stably expressing EGFP (40 µg), Tomato (40 µg), EGFP-MamE (40 µg), or Tomato-MamB (40 µg) were reduced with 100 mM dithiothreitol in sample preparation buffer (188 mM Tris-HCl pH 6.8/2% SDS/0.1% Bromophenol Blue/43% glycerol) and heated at 85 °C for at least 5 min. Reduced samples were then subjected to discontinuous SDS polyacrylamide gel electrophoresis (SDS-PAGE) using a 10% running gel. Protein was transferred onto a nitrocellulose blot using the Original iBlot Gel Transfer Device (Life Technologies, Burlington, ON, Canada).

For EGFP detection, nonspecific protein binding was blocked in 5% bovine serum albumin (BSA)/Tris-buffered saline pH 7.4 (TBS) for 3 h at room temperature. Blots were then incubated for 15 h in 1:1000 mouse α-GFP (Invitrogen)/3% BSA/TBS/0.02% sodium azide (TBSA); then washed using TBS/0.1% Tween 20 (TBST; Sigma-Aldrich, Oakville, ON, Canada) for 30 min with 4 changes of buffer; and incubated for 2 h in 1:20,000 horseradish peroxidase (HRP)-conjugated goat α-mouse IgG (Sigma-Aldrich)/1% BSA/TBS. All incubations were performed at room temperature. Blots were then washed with 0.1% TBST for 30 min with 4 changes of buffer and imaged using the Chemigenius Gel Doc (Syngene, Bengaluru, India). A chemiluminescent signal was detected using SuperSignal West Pico Chemiluminescent Substrate (Thermo Fisher Scientific), according to the manufacturer’s instructions.

For Tomato detection, blots were blocked in 3% BSA/TBSA for approximately 18 h at room temperature and then incubated for 18 h in 1:1000 primary goat α-tdTomato (MyBioSource, San Diego, CA, USA)/3% BSA/TBSA at 4 °C. After washing in 0.1% TBST as described above, blots were incubated for 1 h in 1:20,000 HRP-conjugated rabbit α-goat IgG (Sigma-Aldrich)/1% BSA/TBS at room temperature.

Glyceraldehyde 3-phosphate dehydrogenase (GAPDH) was used as a loading control. For GAPDH detection, blots were placed in stripping solution (63 mM Tris-HCl pH 6.8/2% SDS/0.016% β-mercaptoethanol) and agitated in a 37 °C water bath for 30 min prior to washing in 0.1% TBST and blocking in 5% BSA/TBS. The primary and secondary antibodies were 1:2000 rabbit α-GAPDH (Sigma-Aldrich)/3% BSA/TBSA and 1:20,000 HRP-conjugated goat α-rabbit IgG (Sigma-Aldrich)/1% BSA/TBS, respectively.

### 2.5. Confocal Imaging

Stably transfected cell lines were examined with confocal fluorescence microscopy (using a Nikon A1R confocal microscope; Keyence Canada, Mississauga, ON, Canada) to confirm expression and characterize the intracellular localization of EGFP-MamI, Tomato-MamL, Tomato-MamL_trunc,_ FLAG-MamL, EGFP-MamE (T317A), and Tomato-MamB fusion proteins. In preparation for confocal microscopy, approximately 1 × 10^5^ cells were cultured in a 35 mm glass-bottom dish (MatTek Corporation, Cedarlane, Burlington, ON, Canada) for 48 h. On the day of imaging, the dish was placed in a stage-top incubator to maintain 37 °C and 5% CO_2_. Images and cines were captured using a Galvano scanner with NIS-Elements AR 5.11.01 (Nikon Instruments Inc.), and a 20× air objective with 0.75 numerical aperture and an average pixel width of 0.2 µm/pixel. To capture images of cells expressing a single fluorophore, the FITC microscope filter (495 nm excitation/519 nm emission) was used for cells expressing the EGFP fluorophore and the TRITC microscope filter (557 nm excitation/576 nm emission) was used for cells expressing the Tomato fluorophore. To capture images of cells co-expressing both fluorophores (EGFP and Tomato), the FITC and TRITC filters allowing for simultaneous 495 nm and 557 nm excitations were used. When needed, captured images of cells in both channels were then merged in Adobe Photoshop CS7.

Time-lapses were acquired with the time lapse function in NIS-Elements AR 5.11.01, recording an image every 1 s for a total of 60 s. The duration of movies playing 10-fold faster than real time is 6 s. Cines were captured in either channel or both channels simultaneously, as described above. The NIS-Elements AR 5.11.01 software automatically generated a time lapse video in nd2 format with single or merged channels. This video was then edited in Adobe Photoshop CS7 by cropping and then pasting into a single file, which could be exported in a graphics interchange format (GIF).

### 2.6. Quantification of Cell Phenotypes

Quantification of intracellular fluorescent patterns was performed by visual inspection. Cells were manually counted under the confocal fluorescence microscope and were described based on the fluorescence pattern observed. Every fluorescent cell observed at low magnification was further examined at higher magnification to categorize the pattern. The counts were tallied and the percentages of each fluorescent pattern were calculated.

### 2.7. Microtubule Staining

MDA-MB-435 cells stably expressing Tomato-MamL alone, or co-expressing FLAG-MamL and EGFP-MamI, were cultured on a 35 mm glass bottom dish. When the plate reached 70% confluency, ViaFluor 647 Live Cell Microtubule Stain (Cedarlane)/DMEM (low glucose) was added to the culture according to manufacturer protocol. After 30 min of incubation at 37 °C with 5% CO_2_, the dish was imaged by confocal microscopy. Movies of particle mobility were analyzed using ImageJ version 1.8.0 and Mathematica version 13.1. Trajectory maps showing paths travelled by punctate structures were generated in Mathematica using custom code as described below in Particle Trajectory Analysis.

### 2.8. Colchicine Treatment

MDA-MB-435 cells stably expressing Tomato-MamL alone, or FLAG-MamL and EGFP-MamI, were cultured on a 35 mm glass bottom dish. When the plate reached 70% confluency, cells were imaged by confocal microscopy. After the imaging session, 4 µg/mL colchicine (ThermoScientific)/DMEM (low glucose) was added to the dish of cells and incubated for 45 min at 37 °C with 5% CO_2_, as per company protocol. Following colchicine treatment, cells were imaged again by confocal microscopy. Movies of particle mobility before and after colchicine treatment were analyzed using ImageJ and Mathematica. Five videos of cells were collected at each time point for this analysis.

### 2.9. Particle Tracking

Using a plugin called Mosaic Particle Tracker 2D/3D (version 1.0.1) [24] in the software ImageJ version 1.8.0 [25], the trajectories of particles observed in cells expressing either Tomato-MamL, Tomato-MamL/EGFP-MamI, Tomato-MamL_trunc_, Tomato-MamB, or EGFP-MamE (T317A) were determined from confocal cines, and analyzed. Each cell selected for analysis satisfied the following criteria. (a) In any given field of view, a single fluorescent cell, either alone or in a small cluster of cells, was analyzed providing there was no distracting fluorescence outside the cell. (b) The analyzed cell exhibited a healthy morphology: stellate shape, fully adherent on the culture dish, and displaying mitotic figures. (c) The focal plane chosen for each cell analyzed provided the sharpest focus of intracellular punctate fluorescent structures, with no distracting fluorescence above or below the plane of focus.

Cines converted to GIF files were loaded into ImageJ and prepared for analysis by converting them to greyscale; cropping to reduce their size and retaining only the portion of the movie with a single cell; then optimizing brightness and contrast. This last step does not affect the particle tracking but makes it easier for the user to visualize trajectories. For accurate estimation of diffusion coefficients and velocities, the pixel width d = L/N (where L is the width of the field of view in microns and N is the number of pixels) and time interval between two consecutive frames τ = T/(F − 1) (where T is the total duration of the video and F is the total number of frames) were calculated and added to the image properties in ImageJ.

After launching Mosaic Particle Tracker, the parameters used for particle detection were optimized for each cine: values of the radius (size of the tracked particles), cutoff (threshold intensity value below which detected particles are rejected), and percentile (range of intensities below the maximum intensity in the image for which fluorescent spots are considered to be particles) were manually adjusted to allow the software to detect the most manifest particles in the first image of the movie while not picking up lower-intensity noise speckle. Particle detection for a representative cell is shown in Supplementary Figure S2. For particle linking properties, the link range was set to 3 for all cines. In this way, the software would stop tracking a specific particle if it was absent for 3 consecutive frames. The displacement (maximum displacement allowed for a particle between two consecutive frames), which should be set to at least twice the average displacement of a particle between two frames, was set to 10 pixels. The software provided the total number of trajectories detected, a file containing the information relative to each detected trajectory (i.e., the position of the particle in each frame for which it was detected) and the MSD, <r^2^(t)>, of each detected particle.

### 2.10. Simple Trajectory Analysis

The mobility of each particle [24] was first assessed by fitting the MSD for each trajectory (within Mosaic Particle Tracker) with a simple power-law function:

<r^2^(t)> = 4D_a_ t^α^ (1)

A particle undergoing free Brownian motion should have an MSD close to linear in time—that is, with an exponent α close to 1. Constrained Brownian motion would result in α < 1. In contrast, a particle undergoing directed motion should be characterized by α close to 2. If the motion is mixed, with alternate periods of Brownian and directed motion, then one would expect α to be somewhere between 1 and 2. As explained below, results of the fit of MSD was among the information used to differentiate between direct and Brownian motion. For the first group of particles, D_a_ can be considered to be an apparent diffusion coefficient (note that when α ≠ 1, this quantity does not have the dimension of a diffusion coefficient, as it only represents the diffusion coefficient that would be estimated from the fitted value of the MSD at t = 1 s, which is why it is more accurate to speak of an apparent diffusion coefficient).

### 2.11. Refined Algorithm for Trajectory Analysis

Starting from the position and intensity of the tracked particle in each frame returned by Mosaic Particle Tracker, trajectories were further categorized and analyzed using an algorithm written for Mathematica. The main steps of this algorithm are as follows.

Trajectories were first evaluated for inclusion or exclusion based on the following criteria. Trajectories were rejected when either too short (less than 8 frames) or having a frame-to-frame displacement that was too large: that is, more than about 1 micron between successive frames or more than about 3 pixels between non successive frames, if the particle was not detected for one frame (these numbers were adjusted for each cell depending on the imaging parameters). Trajectories were also rejected if the average intensity of the particle was too low (less than one standard deviation below the mean intensity for all particles) or was detected for less than 70% of the trajectory duration.

For each of the remaining trajectories, the MSD was calculated and fitted (for lag times between 1 and 10 s) using Equation (1). The velocity autocorrelation function (VAF), <v(0)v(t)> was also calculated for each of the remaining trajectories, in order to detect potential persistence in the direction of motion (examples of VAF are shown in the Supplementary Data). An estimate of the particle maximum velocity was obtained by identifying the maximum value of the correlation between two successive measurements of the particle apparent velocity:

(2) √(v→(t)·v→(t+τ))

in which τ is the time interval between consecutive frames.

For each cell, a single distribution of displacements was generated for all trajectories in that cell, considering displacements between two successive frames (corresponding to a time interval τ = 1 s). Particles undergoing two-dimensional Brownian diffusion, when sampled at regular time intervals τ, exhibit a distribution of step sizes r described by a Rayleigh distribution: $p\left(r\right)=r/(2D\tau )e^{-r^{2}/4D\tau }$, where D is the diffusion coefficient of the particles [26,27]. Here, to account for the presence of two populations characterized by apparent diffusion coefficients $D_{1}$ and $D_{2}$ and relative fractions $f_{1}$ and $f_{2}$, the displacement distributions obtained for each cell were fitted with the sum of two Rayleigh’s distributions:

(3) $$p\left(r\right)=\frac{f_{1}}{{s_{1}}^{2}}*r*e^{\frac{{-r}^{2}}{{{2s}_{1}}^{2}}}+\frac{f_{2}}{{s_{2}}^{2}}*r*e^{\frac{{-r}^{2}}{{{2s}_{2}}^{2}}}$$

Each term in this expression represents a separate population (i = 1 or 2) characterized by its average step size $s_{i}=\sqrt{2D_{i}\tau }$ and its relative fraction $f_{i}$. In some cases, a third term (a Gaussian peak attributed to steps taken during directed motion) was needed in order to account for a small fraction of longer displacement.

The values of s_1_ and s_2_ (in order of increasing length) became the basis for sorting each individual trajectory into either an immobile, directed, or diffusive trajectory. The criteria used were as follows. (1) For immobile trajectories, the total displacement and largest step are both less than 12 × s_1_. This is based on the reasoning that for immobile particles (population 1), apparent displacements are detected because of localization errors [28], and thus s_1_ corresponds to the precision of the localization procedure (found to be on the order of 20 nm in our experiments). Choosing a limit value for both individual step and total displacements of 12 × s_1_ (on the order of 240 nm) accounts for the possibility that whilst immobile, the particle might apparently take occasional steps larger than s_1_ (for example due to an underlying overall small shift in cell position) and at the same time ensures that the considered particle remains within the same diffraction limited area for the total observation time (of up to 60 s). (2) For a directed trajectory, the total displacement is more than 1.5 × s_2_ × √(n), where n is the number of steps in the trajectory, or the trajectory MSD is characterized by an exponent α > 1.1, as explained above in the section on simple trajectory analysis. This criterion is based on the reasoning that s_2_ (found to be on the order of 100 nm in our experiments) is the average step size of diffusing particles (population 2), thus their average displacement after n steps is s_2_ × √(n). A displacement of 1.5 × s_2_ × √(n) thus points to the particle having a directed motion. (3) Diffusive trajectories are those that are neither immobile nor directed.

### 2.12. Statistical Analysis

All statistical tests were performed using GraphPad Prism version 8. A one-way analysis of variance (ANOVA) with a Tukey’s post hoc test was performed to determine any statistical significance between the apparent diffusion coefficient, velocity, and anomalous exponent values of the trajectories of magnetosome expression systems.

## 3. Results

The following subsections describe the intracellular localization of essential magnetosome proteins as punctate structures, when expressed as fluorescent fusion proteins in a human melanoma cell line. The intracellular mobility of these structures is captured by confocal microscopy in movies and characterized according to particle trajectory, firstly using ImageJ software and a simple power law function, and latterly refined using a custom Mathematica algorithm.

### 3.1. Cellular Distribution of MamE and MamB

**Figure 1:**
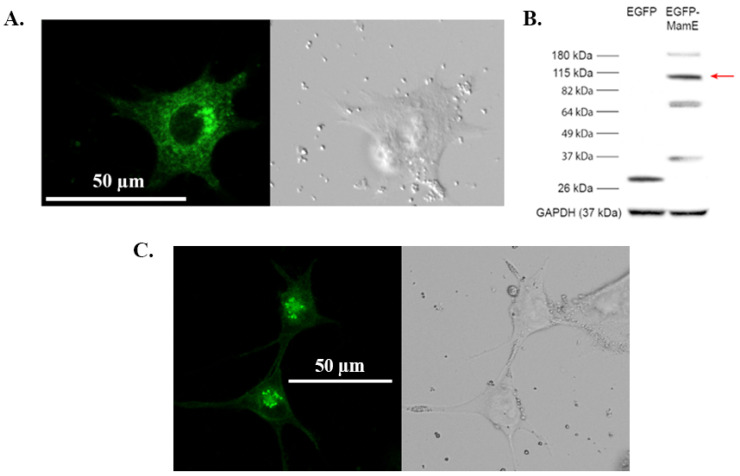
Expression of EGFP-MamE fusion proteins in mammalian cells. (**A**). Confocal fluorescence microscopy of MDA-MB-435 cells stably expressing EGFP-MamE (G49S, T641S) reveals punctate intracellular fluorescence that clusters in the perinuclear region. (**B**). Total cellular protein from cells stably expressing either EGFP or EGFP-MamE (G49S, T641S) were examined by Western blot using a mouse α-EGFP primary antibody. The approximate size of EGFP-MamE is 110 kDa (red arrow). Bands at lower molecular weights are consistent with MamE autoproteolysis [30]. Approximate MW is shown in the left margin. The bottom panel shows the loading control, GAPDH. (**C**). Confocal fluorescence microscopy of MDA-MB-435 cells stably expressing EGFP-MamE (T317A) also displays a punctate intracellular fluorescence pattern. MamE particles are not evidently mobile as shown in Video S1.

When the magnetosome protein MamE is compared across MTB species [8], its sequence consists of both conserved and variable regions [21,29] (Supplementary Figure S1). To address the influence of sequence variability in mammalian cell expression systems, we compared two EGFP-MamE fusion proteins, representing 3 distinct point mutations relative to the consensus sequence (Supplementary Figure S1). Potential differences in their intracellular expression in MDA-MB-435 cells were examined using confocal fluorescence microscopy. Cells stably expressing EGFP-*mamE* (G49S, T641S) display intracellular fluorescence in a punctate pattern with a diffuse fluorescence background (Figure 1A). These EGFP-MamE structures are numerous and cluster near the nucleus. Protein expression was confirmed by immunoblot (Figure 1B and Supplementary Figure S3) and reveals potential autoproteolysis of MamE as previously reported in MTB [30,31]. Unlike the expression of MamL [19] or MamB (described below), MamE-expressing particles do not display mobility on visual inspection. Figure 1C shows that cells expressing EGFP-*mamE* (T317A) display a similar expression pattern. Confocal fluorescence microscopy reveals a distinct intracellular, punctate green fluorescence that clusters near the nucleus. Similar to EGFP-MamE (G49S, T641S), the expression of EGFP-MamE (T317A) also exhibits a diffuse background and little or no mobility on visual inspection. Additionally, the effect of both EGFP-MamE (G49S, T641S) and EGFP-MamE (T317A) on cellular magnetic resonance relaxation rates were evaluated and no differences were found between the two mutants [18].

**Figure 2:**
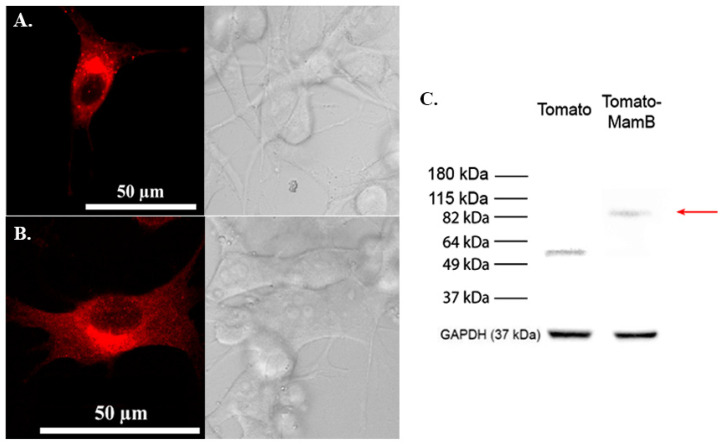
Expression of Tomato-MamB fusion protein in mammalian cells. Confocal fluorescence microscopy of MDA-MB-435 cells stably expressing Tomato-MamB displays either punctate intracellular fluorescence (**A**) or diffuse fluorescence (**B**). The punctate structures in (**A**) are dispersed throughout the cell. Total cellular protein from cells stably expressing either Tomato or Tomato-MamB were examined by Western blot (**C**) using a rabbit α-Tomato primary antibody. The approximate size of Tomato-MamB is 91 kDa (red arrow). Approximate MW is shown in the left margin. The bottom panel shows the loading control, GAPDH. MamB particles are mobile as shown in Video S2.

When Tomato-MamB is stably expressed in MDA-MB-435 cells, red fluorescence is displayed in a punctate pattern in the majority of the transfected cell population (~70%; Figure 2A) and in a diffuse pattern in about 30% of the transfected cell population (Figure 2B). Importantly, these punctate MamB structures are mobile. Protein expression was confirmed by immunoblot (Figure 2C and Supplementary Figure S4).

### 3.2. Analysis of EGFP-MamE Trajectories

**Figure 3:**
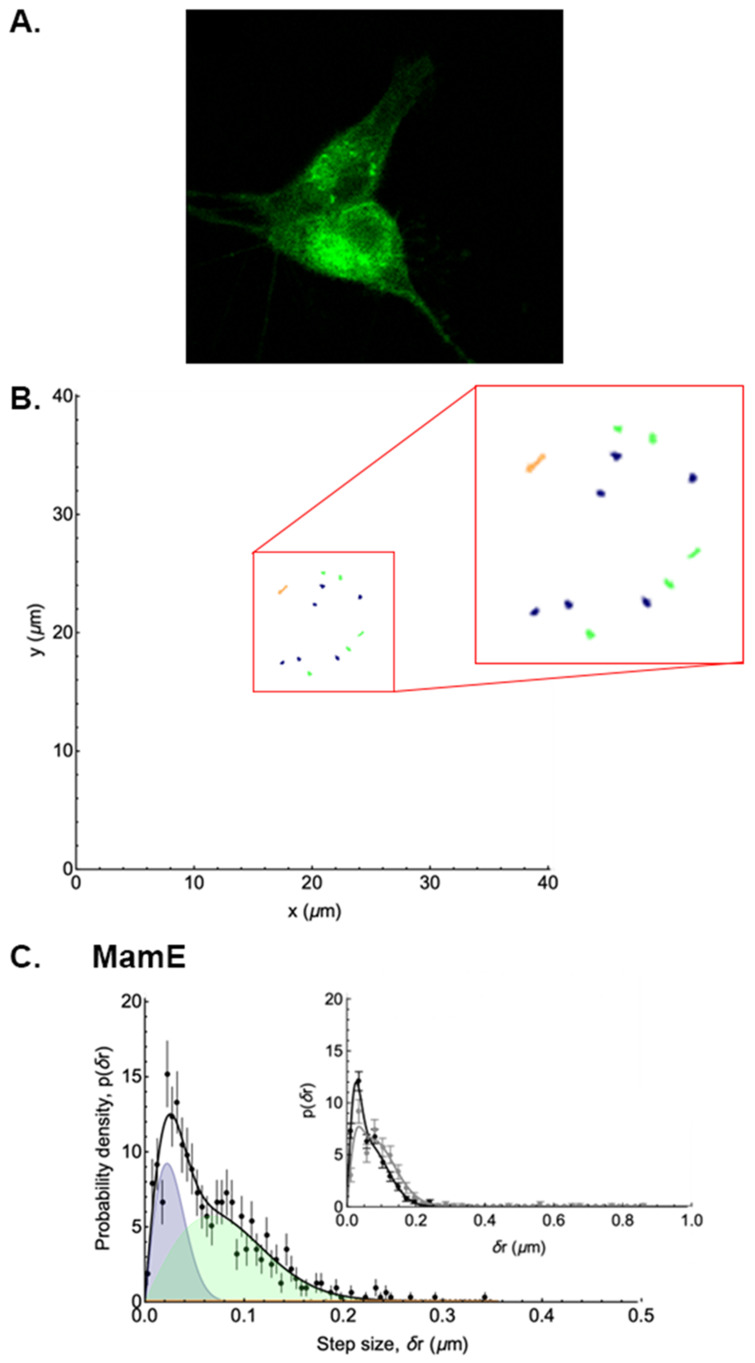
Distribution of the displacements of punctate structures in a representative EGFP-MamE-expressing mammalian cell. The analysis of punctate structures in the confocal micrograph of a representative MamE-expressing cell (**A**) reveals 3 types of trajectories (**B**) with stationary particles shown in dark blue, and Brownian motion and directed motion trajectories represented in green and orange, respectively. The inset enlarges these restricted trajectories 2-fold. The probability density of displacements (**C**) after t = 1 s is shown in the main panel (black symbols) and fitted assuming two diffusive populations and one directed population. The blue and green shaded peaks represent the contributions of the immobile and Brownian diffusing populations, respectively, while the orange shaded peak—barely visible in this case—is that of the directed population. The black curve shows the sum of these three contributions. In the inset, the sum of these three contributions after 1 s (black symbols) is compared to the sum after 20 s (grey symbols) and shows very little difference in the distribution of displacements over time.

As no evident motion of the puncta associated with MamE protein was observed in confocal movies (refer to Video S1 provided in both real time and 10× faster), the analysis of EGFP-*mamE* (T317A)-expressing cells was chosen to serve as an example of cells containing particles with low mobility. Using this reference point, the signature of these MamE particles is contrasted with that of the higher mobility particles observed in other expression systems. A visual representation of MamE trajectories and the distribution of their displacement is shown in Figure 3 (and Supplementary Figure S5). It is apparent from this distribution that the motion of MamE particles is very highly constrained, since almost no single displacement after t = 1 s is larger than 0.2 µm, and since the displacements after 20 s are almost the same as after 1 s. Trajectory analysis of MamE fluorescent structures showed that 26% of these particles were immobile, 62% were undergoing Brownian motion, and only 13% underwent directed motion at some point in their trajectories (Table 2). According to this analysis, the average apparent diffusion coefficient of MamE particles undergoing Brownian motion is 1.9 ± 0.4 × 10^−3^ µm^2^/s. The average velocity of the very few MamE particles undergoing directed motion is 0.17 ± 0.03 µm/s.

### 3.3. Mobility of Tomato-MamB

**Figure 4:**
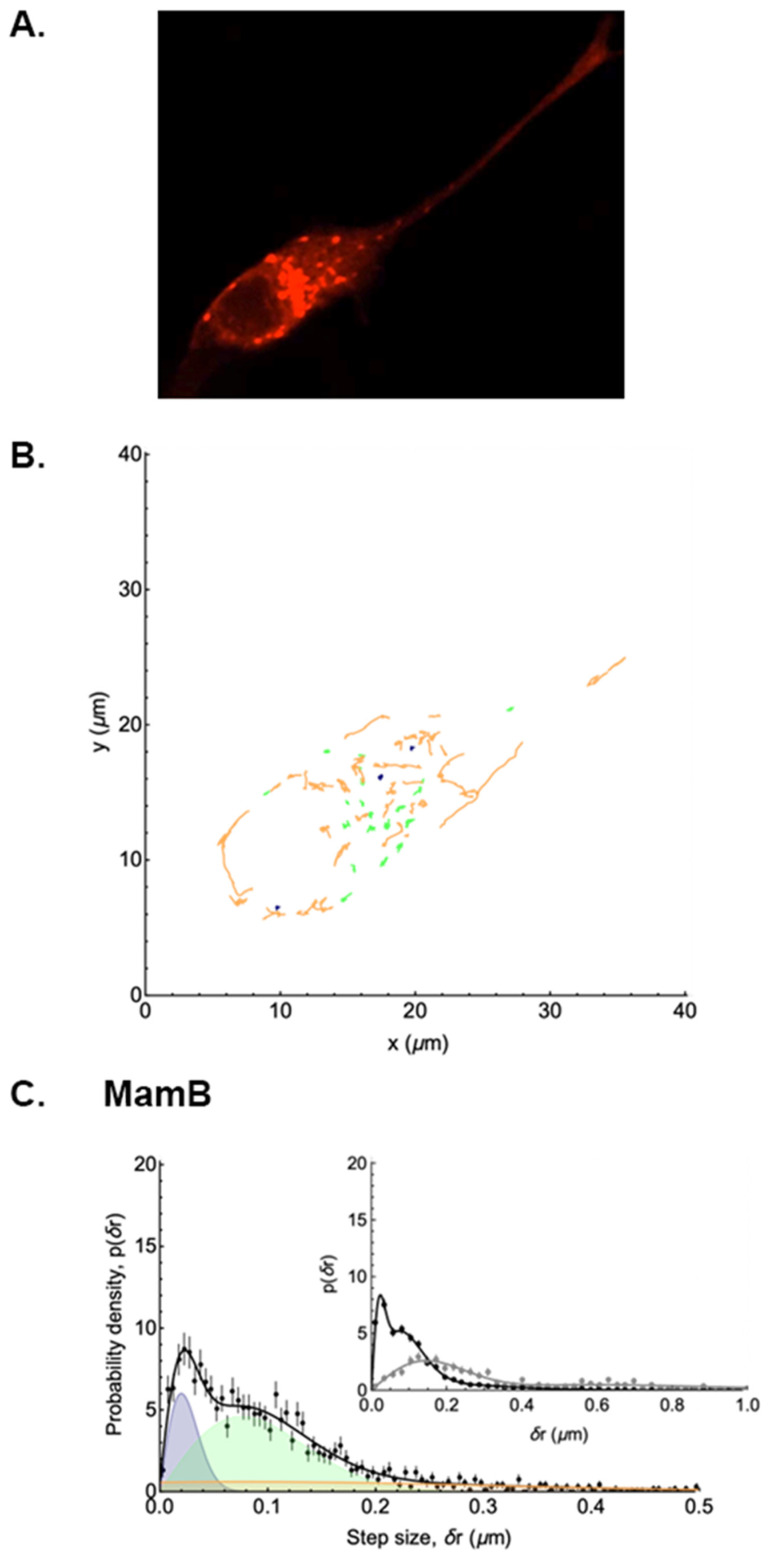
Distribution of the displacements of punctate structures in a representative Tomato-MamB-expressing mammalian cell. The analysis of punctate structures in the confocal micrograph of a representative MamB-expressing cell (**A**) reveals 3 types of trajectories (**B**) with stationary particles shown in dark blue, and Brownian motion and directed motion trajectories represented in green and orange, respectively. The probability density of displacements (**C**) after t = 1 s is shown in the main panel (black symbols) and fitted assuming two diffusive populations and one directed population. The blue and green shaded peaks represent the contributions of the slow and fast diffusing populations, respectively, while the orange shaded peak is that of the directed population. The black curve shows the sum of these three contributions. In the inset, the sum of these three contributions after 1 s (black symbols) is compared to the sum after 20 s (grey symbols), and shows a broad distribution of displacements over time.

Analyses of Tomato-MamB particle trajectories also reveal both directed and Brownian motion in each cell, with the occurrence of directed trajectories markedly greater than in MamE-expressing cells. A visual representation of the detected MamB particles (Video S2 and Figure 4A) and analyzed trajectories (Figure 4B) is provided alongside their displacement distribution (Figure 4C and Supplementary Figure S6). The difference between MamE and MamB is clearly shown by the large number of displacements after t = 1 s between 0.2–0.4 µm and by the broad distribution of displacements after 20 s (Figure 4C inset), as expected for mobile particles. Analysis of MamB trajectories showed that only 15% of MamB particles were immobile, while 57% were undergoing Brownian motion, and 28% underwent directed motion at some point in their trajectories (Table 2). The average apparent diffusion coefficient of MamB particles undergoing Brownian motion is 4.0 ± 1.0 × 10^−3^ µm^2^/s and 2-fold greater than MamE particles (*p* < 0.05). The average velocity of MamB particles undergoing directed motion is 0.24 ± 0.01 µm/s.

### 3.4. Mobility of Tomato-MamL

**Figure 5:**
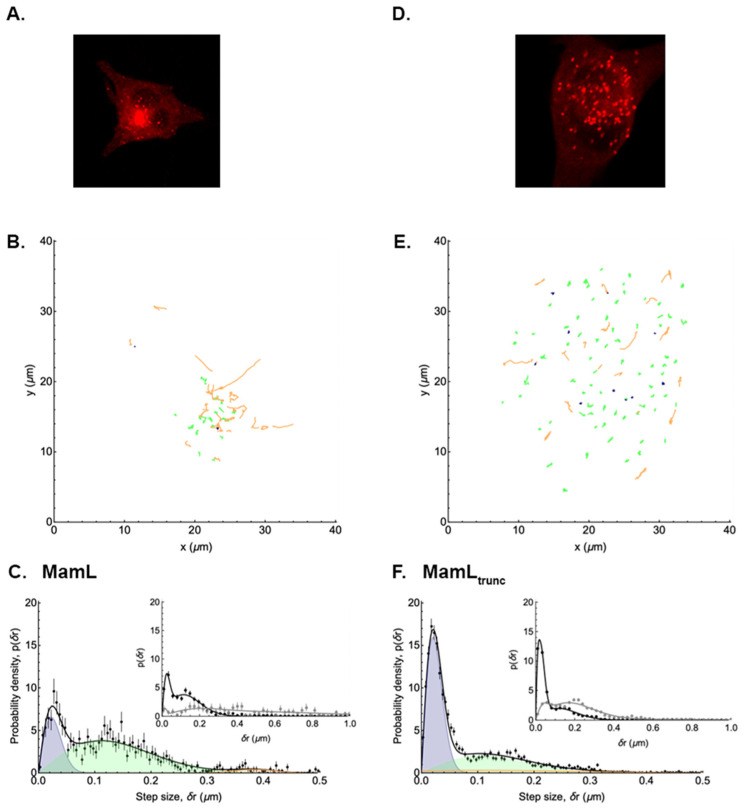
Comparing the displacement distributions of punctate structures in representative Tomato-MamL- and Tomato-MamL_trunc_-expressing mammalian cells. The analysis of punctate structures in the confocal micrograph of a representative Tomato-MamL-expressing cell (**A**) reveals 3 types of trajectories (**B**) with stationary particles shown in dark blue, and Brownian motion and directed motion trajectories represented in green and orange, respectively. The probability density of displacements (**C**) after t = 1 s is shown in the main panel (black symbols) and fitted assuming two diffusive populations and one directed population. The blue and green shaded peaks represent the contributions of the slow and fast diffusing populations, respectively, while the orange shaded peak is that of the directed population. The black curve shows the sum of these three contributions. In the inset, the sum of these three contributions after 1 s (black symbols) is compared to the sum after 20 s (grey symbols) and shows a broad distribution of displacements over time. These data are compared to a representative Tomato-MamL_trunc_-expressing cell (**D**) and the displacement of its punctate structures (**E**). Once again, the probability density of displacements (**F**) after t = 1 s is shown in the main panel (black symbols) and fitted assuming two diffusive populations and one directed population. The blue and green shaded peaks represent the contributions of the slow and fast diffusing populations, respectively, while the orange shaded peak is that of the directed population. The black curve shows the sum of these three contributions. In the inset, the sum of these three contributions after 1 s (black symbols) is compared to the sum after 20 s (grey symbols) and shows a persistent broad distribution of displacements over time, despite removal of the MamL C-terminal peptide.

Previous characterization of Tomato-MamL particles [19] revealed patterns of mobility similar to those of Tomato-MamB. A visual representation of detected Tomato-MamL particles and analyzed trajectories is shown in Figure 5A,B, respectively. The distribution of their displacements is plotted in Figure 5C and Supplementary Figure S7, with a broad distribution apparent after 20 s. Analysis of these results showed that 18% of MamL particles were immobile; 56% were undergoing Brownian motion; and 25% underwent directed motion at some point in their trajectories (Table 2) as seen for MamB-expressing particles. The average apparent diffusion coefficient of MamL particles undergoing Brownian motion is 5.1 ± 1.7 × 10^−3^ µm^2^/s. The average velocity of MamL particles undergoing directed motion is 0.19 ± 0.05 µm/s. Similar to Tomato-MamB, the apparent diffusion coefficient of Tomato-MamL particles is over 2-fold greater than EGFP-MamE (*p* < 0.001, Table 2) while the velocity of any one particle remains approximately the same between expression systems (Table 2).

To better understand the mobility of MamL, we introduced an early stop codon to remove 15 amino acids from the C-terminus of the MamL protein and examined this truncated form. The C-terminal peptide that was removed may interact with mobile cytoskeletal elements based on sequence similarity to cell-penetrating peptides [6,21]. A visual representation of the detected MamL_trunc_ particles and analyzed trajectories is shown in Figure 5D,E, respectively, with their displacement distribution plotted in Figure 5F (and Supplementary Figure S8). Despite truncation of its C-terminal peptide, there remains a broad distribution of displacements after t = 20 s (inset). Analysis of MamL_trunc_ trajectories showed that 14% of MamL_trunc_ particles were immobile, 60% were undergoing Brownian motion, and 26% underwent directed motion at some point in their trajectories (Table 2). While the distribution of particles is similar to native MamL, the average apparent diffusion coefficient of MamL_trunc_ particles undergoing Brownian motion was reduced more than 2.5-fold (*p* < 0.001) to 1.9 ± 1.1 × 10^−3^ µm^2^/s (Table 2). The average velocity of MamL_trunc_ particles undergoing directed motion is 0.14 ± 0.06 µm/s and comparable to all other particles expressing magnetosome proteins (Table 2).

### 3.5. Mobility of Co-Expressed Tomato-MamL/EGFP-MamI

**Figure 6:**
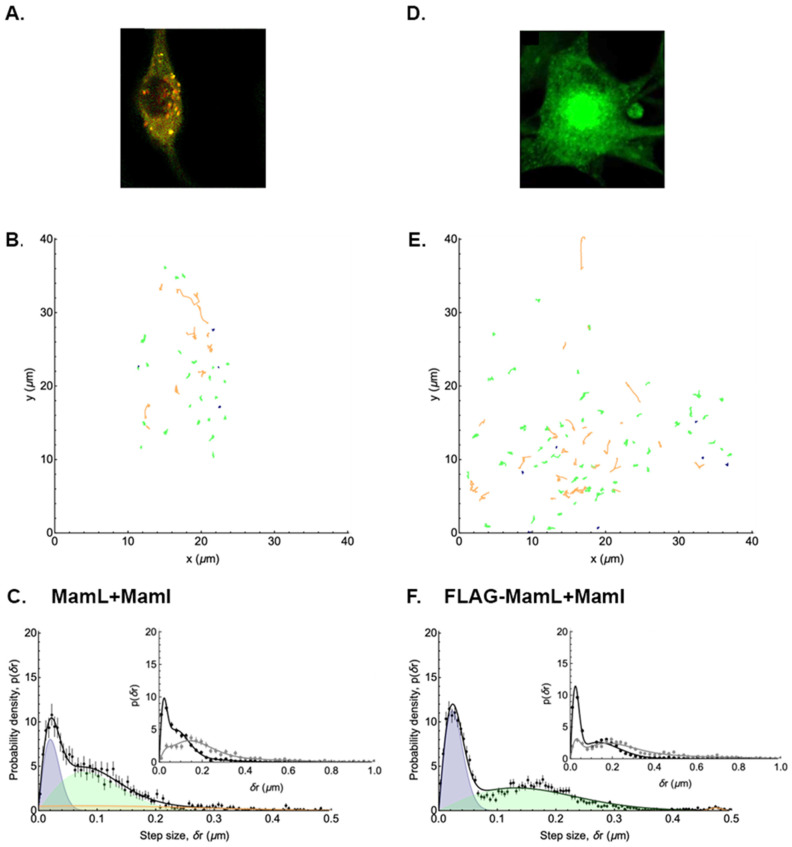
Comparing the displacement distributions of punctate structures in representative mammalian cells co-expressing Tomato-MamL + EGFP-MamI or FLAG-MamL + EGFP-MamI. The analysis of punctate structures in the confocal micrograph of a representative cell co-expressing Tomato-MamL + EGFP-MamI ((**A**), yellow fluorescence) reveals 3 types of trajectories (**B**) with stationary particles shown in dark blue, and Brownian motion and directed motion trajectories represented in green and orange, respectively. The probability density of displacements (**C**) after t = 1 s is shown in the main panel (black symbols) and fitted assuming two diffusive populations and one directed population. The blue and green shaded peaks represent the contributions of the slow and fast diffusing populations, respectively, while the orange shaded peak is that of the directed population. The black curve shows the sum of these three contributions. In the inset, the sum of these three contributions after 1 s (black symbols) is compared to the sum after 20 s (grey symbols), and shows a broad distribution of displacements over time. By replacing Tomato with a FLAG sequence, a comparison is drawn to representative data from FLAG-MamL + EGFP-MamI co-expression (**D**), green fluorescence) and the displacement of these punctate structures (**E**). The probability density of displacements (**F**) after t = 1 s is shown in the main panel (black symbols) and fitted assuming two diffusive populations and one directed population. As before, the blue and green shaded peaks represent the contributions of the slow and fast diffusing populations, respectively, while the orange shaded peak is that of the directed population. The black curve shows the sum of these three contributions. In the inset, the sum of these three contributions after 1 s (black symbols) is compared to the sum after 20 s (grey symbols) and shows a broadening distribution of displacements over time, whether or not MamL is fused to Tomato.

Tomato-MamL particles not only retain their mobility when co-expressed with EGFP-MamI, the latter appear to be recruited to the same mobile complex [19]. These mobile particles, consisting of co-localized protein, also display both directed and Brownian motion. A visual representation of the detected MamL + MamI particles and trajectories is shown in Figure 6A,B, respectively, with their displacement distribution plotted in Figure 6C (and Supplementary Figure S9). Similar to the mobility of particles in other MamL expression systems, there is a broad distribution after t = 20 s (inset). Analysis of the MamL + MamI trajectories revealed that 21% of MamL + MamI particles were immobile, 45% were undergoing Brownian motion, and 32% underwent directed motion at some point in their trajectories (Table 2). Compared to particles expressing MamE, these data suggest that co-expression of essential magnetosome proteins more than doubles the fraction of particles that undergo directed motion. Thus, much like MamL and MamB alone, mobility is preserved even when MamL is interacting with MamI [19]. The average apparent diffusion coefficient of MamL + MamI particles undergoing Brownian motion is 3.2 ± 2.5 × 10^−3^ µm^2^/s. The average velocity of MamL + MamI particles undergoing directed motion is 0.23 ± 0.09 µm/s, consistent with the other expression systems examined (Table 2).

A FLAG-MamL/EGFP-MamI co-expression system was created to simplify confocal image analysis using a single chromophore (EGFP) and to remove bulky fluorescent proteins attached to MamL (i.e., tandem Tomato) that may compromise magnetosome protein interactions [19]. The trajectories of particles from stable FLAG-MamL/EGFP-MamI co-expression were analyzed and compared to those of the dual fluorescent Tomato-MamL/EGFP-MamI particles. A visual representation of detected FLAG-MamL + MamI particles and their displacements is shown in Figure 6D,E, respectively, with the displacement distribution plotted in Figure 6F and Supplementary Figure S10. Similar to Tomato-MamL/EGFP-MamI, the analysis of FLAG-MamL/EGFP-MamI trajectories showed that a minority of the particles were immobile (13%) while the majority of particles were undergoing Brownian motion (66%) and directed motion (21%) at some point in their trajectories (Table 2). The average apparent diffusion coefficient of FLAG-MamL + MamI particles undergoing Brownian motion is 5.0 ± 0.9 × 10^−3^ µm^2^/s (Table 2). The average velocity of FLAG-MamL + MamI particles undergoing directed motion is 0.14 ± 0.05 µm/s and once again comparable to the other particles formed by the essential magnetosome proteins examined (Table 2). Hence, the use of EGFP alone to monitor the mobility of MamL-MamI interactions does not alter trajectory analysis to any appreciable degree.

### 3.6. Interaction of Magnetosome Proteins with Microtubules

**Figure 7:**
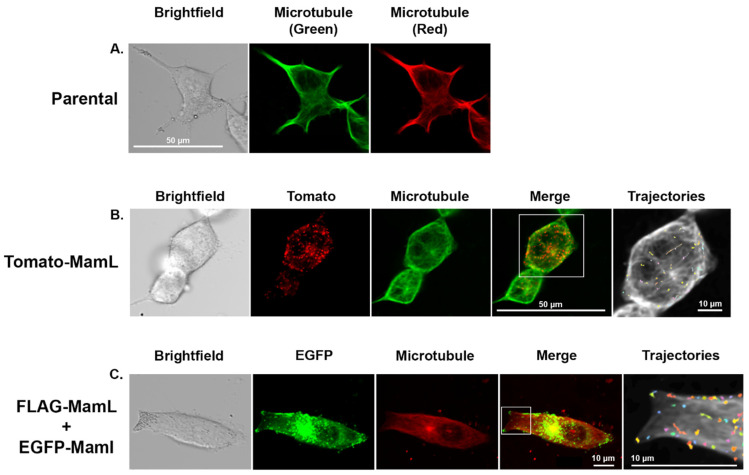
Microtubule staining of MDA-MB-435 cells. ViaFluor 647 Live Cell Microtubule Stain was used to treat MDA-MB-435 parental cells (**A**), MDA-MB-435 cells stably expressing Tomato-MamL (**B**), or MDA-MB-435 cells co-expressing FLAG-MamL and EGFP-MamI (**C**). Microtubule images were captured in the Cy5 channel, which is manually coloured red or green depending on expression of the fluorescent fusion protein (EGFP-MamI or Tomato-MamL, respectively). The “merge” panels show punctate magnetosome fusion proteins overlayed on the cell’s microtubule network. A white box outlines the “trajectories” panel showing the paths travelled by punctate structures overlayed on a black-and-white contrast-enhanced image of the cell’s microtubule network. MamL, alone or interacting with EGFP-MamI, displays mobility along microtubules.

Since directed motion in live cells is often associated with molecular motors transporting cargos along filaments, the coincidence of directed trajectories with microtubule networks was investigated. Live cell staining of MDA-MB-435 microtubules reveals a structured network of filaments throughout the cell and external to the nuclear membrane (Figure 7A). When cells expressing Tomato-MamL were stained, imaging and trajectory analysis revealed that MamL particles were travelling along the microtubule paths (Figure 7B, Video S3 and Supplementary Figure S11). Microtubule staining of MDA-MB-435 cells co-expressing EGFP-MamI and FLAG-MamL reveals a similar result, with MamL + MamI particles travelling along microtubules (Figure 7C, Video S4 and Supplementary Figure S11). Trajectory analysis after live cell staining indicates that MamL particles move at 0.16 µm/s and MamL + MamI particles move at 0.24 µm/s, consistent with no appreciable effect of microtubule staining on particle mobility (Supplementary Table S1).

To further determine whether magnetosome proteins interact with the cell’s cytoskeleton, colchicine was used to treat MDA-MB-435 cells expressing Tomato-MamL or co-expressing FLAG-MamL + EGFP-MamI. By binding to tubulin, colchicine destabilizes microtubules and thus also interferes with intracellular mobility. Additionally, colchicine affects actin polymerization [32] and has been shown to bind and disrupt actin filaments [33]. Table 3 shows that disruption of the cytoskeleton reduced the fraction of Tomato-MamL particles undergoing directed motion by approximately fivefold, without significantly altering maximum velocity or apparent diffusion coefficient. However, the analysis of particles co-expressing FLAG-MamL + EGFP-MamI showed significant decreases in maximum velocity (from 0.14 ± 0.03 µm/s to 0.05 ± 0.02 µm/s, *p* < 0.001) and apparent diffusion coefficient (from 4.6 ± 2.4 µm^2^/s to 1.8 ± 0.4 µm^2^/s, *p* < 0.05) upon colchicine treatment. MamL particles undergoing Brownian motion were not significantly disrupted by colchicine treatment (from 5.1 ± 1.7 × 10^−3^ µm^2^/s to 4.3 ± 0.8 × 10^−3^ µm^2^/s). Representative trajectory analysis of these data is shown in Supplementary Figures S12 and S13.

## 4. Discussion

This is the first report to describe the stable expression of MamE and MamB in mammalian cells and to analyze the intracellular mobility of punctate structures formed by essential magnetosome proteins expressed in this eukaryotic environment. For each fluorescent fusion protein analyzed (MamL, MamL_trunc_, MamL + MamI, MamB, or MamE), three types of particle movement were observed for the puncta: confined trajectories, Brownian trajectories, and trajectories with stretches of directed motion.

When associated with magnetosome proteins, particles undergoing Brownian motion have apparent diffusion coefficients between 0.002 and 0.005 µm^2^/s. This diffusion coefficient is very slow compared to that of a soluble protein in the cytoplasm of mammalian cells, which is on the order of 10 µm^2^/s [34]. Assuming that MamI, MamL, MamB, and MamE are all membrane associated proteins when expressed in mammalian cells, as in MTB, the measured diffusion coefficients and the brightness of the observed particles [19] are consistent with the notion that these magnetosome proteins are part of a rather large (membranous) structure containing many such proteins and not simply part of the soluble protein fraction. Since these punctate structures cannot be resolved by confocal microscopy, the approximate radius of these fluorescent particles is expected to be less than 300 nm, i.e., smaller than the resolution of the microscope ($\sim \frac{\lambda }{2NA}=\frac{500}{1.5}$). Hence, the possibilities for particles of this diameter include localization in the membrane of pre-existing lipid vesicles [35], such as lysosome [36] and/or peroxisomes [37], or a role in the formation of new ones. Confined trajectories may thus correspond to particles localized in immobilized vesicles.

When associated with magnetosome proteins, particles that undergo active directed motion have a maximum velocity from 0.1–0.3 µm/s. These particles usually also undergo stretches of Brownian motion or immobility. The fluorescent magnetosome fusion proteins may be localized in vesicles that are attached to molecular motors and/or may be directly interacting with molecular motors as they transiently walk along protein filaments.

### 4.1. Brownian Motion of Magnetosome Proteins

The majority of MamE particles are either immobile (26%) or undergoing some form of Brownian motion (62%); however, several features of this analysis point toward a very restricted type of Brownian motion. First, Brownian MamE particles have an apparent diffusion coefficient of 1.9 ± 0.4 × 10^−3^ µm^2^/s, which is significantly lower than the diffusion of MamL and MamB particles. This suggests that MamE is either localized in different structures than MamL and MamB, with more restricted mobility or that MamE expression leads to immobilization of the structures that may also contain MamL and MamB. The possible co-localization of MamL, MamB and MamE is under examination [38]. Second, Brownian MamE particles have trajectories characterized by an extremely low anomalous exponent of 0.15 ± 0.04, the lowest α value of all the magnetosome protein expression systems examined. An α value below 1 implies constrained or restricted diffusion (i.e., diffusion in the presence of obstacles), and a value below 0.5 suggesting caged diffusion [39,40]. Taken together, the low diffusion coefficient of MamE particles, extremely low value of the anomalous exponent α, and the high occurrence of relatively stationary MamE particles suggest that this protein is the most constrained out of the magnetosome proteins studied in mammalian cells.

In contrast to MamE, Brownian MamL and MamB particles have a noticeably larger diffusion coefficient (on the order of 4–5 × 10^−3^ µm^2^/s), and a larger anomalous exponent α (on the order of 0.4 to 0.5). These values are consistent with magnetosome proteins localizing on particles (e.g., lipid vesicles) that are tens to hundreds of nm in size and undergoing restricted diffusion [36]. By removing the MamL C-terminal to create MamL_trunc_ particles, diffusion was significantly reduced compared to full-length MamL particles (*p* < 0.001). The lack of MamL C-terminal peptide may cause the protein to localize in different (more confined) structures than those formed by full-length MamL. Alternatively, the MamL C-terminal may confer mobility, either through its association with the particle structure or by binding directly to molecular motors. Future research is warranted to address these interesting possibilities.

When MamL is co-expressed with MamI, the diffusion coefficient of Tomato-MamL/EGFP-MamI particles (3.2 ± 2.5 × 10^−3^ µm^2^/s) is not significantly different from the diffusion of structures with MamL alone (5.1 ± 1.7 × 10^−3^ µm^2^/s). This is consistent with the localization of MamL and MamI within the same structures/particles, and that MamI is recruited to the same structure as MamL when they interact [19]. This finding is further supported by the expression of FLAG-MamL/EGFP-MamI. Those particles diffuse at 5.0 ± 0.9 × 10^−3^ µm^2^/s, no different than the apparent diffusion coefficients of other MamL-containing particles. Furthermore, the α value of Tomato-MamL alone, Tomato-MamL/EGFP-MamI, and FLAG-MamL/EGFP-MamI particles are all comparable, consistent with MamL and MamI colocalization on a similar structure.

### 4.2. Directed Motion of Magnetosome Proteins

**Figure 8:**
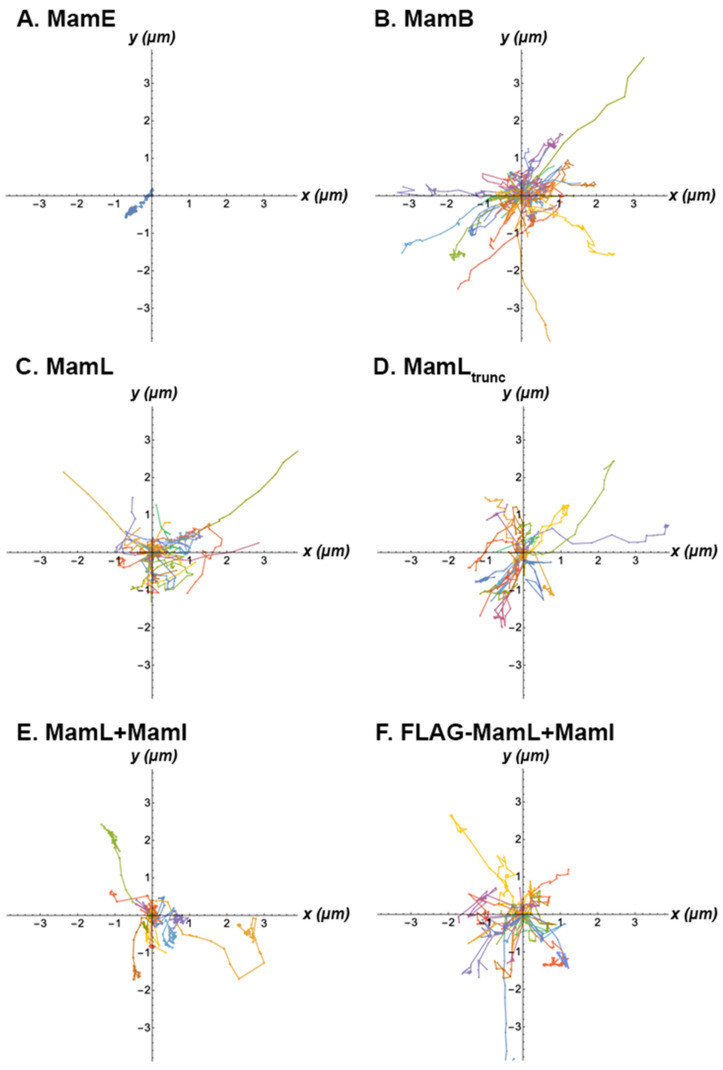
Representative directed trajectories of magnetosome proteins expressed in mammalian cells. Each panel shows all the directed trajectories detected in a single representative cell, for each of the expression systems under study: (**A**) EGFP-MamE, (**B**) Tomato-MamB, (**C**) Tomato-MamL, (**D**) Tomato-MamL_trunc_, (**E**) Tomato-MamL and EGFP-MamI, and (**F**) FLAG-MamL and EGFP-MamI. All trajectories have been plotted by placing the first point of the trajectory at the point with coordinates x = y = 0. Each coloured line represents the distance and direction travelled by a single trajectory.

Although the magnetosome proteins studied undergo directed motion to varying degrees (i.e., variability in the percentage of detected particles that display directed motion and in the average value of the α exponent for these directed trajectories; Supplementary Table S2), similar velocities (mean value of 0.19 ± 0.06 µm/s) were measured for all particles. In addition, except for MamE particles, the aspect of these directed trajectories (which very often are accompanied by a period of intermittent Brownian motion) is similar for all studied proteins (Figure 8).

The mammalian cytoskeleton is composed of various filaments such as actin and microtubules [41]. These filamentous components cross-regulate each other [42] and are responsible for membrane bending, cargo transport, cell division, and various other cytoskeletal regulations [41]. When tracking punctate structures along the microtubular network, the observed directed motion of magnetosome fluorescent fusion proteins suggests that detected particles interact with a linear molecular motor, for example a type of kinesin or dynein subunit. This interaction is further supported by the disruption of intracellular motion in the presence of colchicine. Kinesin and dynein are motor proteins that both travel along microtubules to move cargo (i.e., vesicles and organelles) to and from the cell’s periphery, respectively [43]. Studies on mammalian molecular motors report kinesin velocities ranging from 0.1 to 0.8 µm/s and dynein velocities from 0.075 to 0.85 µm/s [44]. In addition, an interaction between magnetosome fluorescent fusion proteins and molecular motors associated with actin filaments is implied, for example a type of myosin subunit. In mammalian cells, the myosin family constitutes a group of molecular motors responsible for directed movement of intracellular cargo along actin filaments. These structures, specifically myosin IB, myosin II, and myosin V, all move at a velocity of 0.2 µm/s [45]. The speeds observed for the detected particles are therefore consistent with interactions with myosin. In MTB, others have reported interactions of the magnetosome chain with MamK, an actin-like magnetosome protein [46,47] and MamJ, a filamentous protein responsible for magnetosome chain assembly [46]. Thus, MamK-MamJ interactions anchor magnetosomes to cytoskeletal elements and regulate the dynamic movement of these bacterial organelles. As observed in mammalian cells, the mobility of select magnetosome proteins reflect features of magnetosome cytoskeletal interactions in MTB. These findings are consistent with the potential formation of a rudimentary magnetosome-like nanoparticle.

MamE stands out from other magnetosome proteins studied here in that many fewer MamE particles are undergoing directed motion and/or they spend less time undergoing directed motion (Figure 8). This is further indicated by the low α exponent of 0.26 ± 0.32. Particles undergoing directed motion should have α exponents close to 2; however, a lower exponent can also indicate that the directed motion alternates with either Brownian motion or immobilized motion, as is the case with all particles detected and especially those associated with MamE. When undergoing directed motion, MamE particles move at a velocity of 0.17 µm/s. From protein structure analysis [48,49], the cytoplasmic C-terminal polypeptide of MamE is short and has only 2 positively-charged amino acids; therefore, MamE most likely does not bind to molecular motors (which hold a negative charge [20,21]) strongly or frequently due to the small positive charge. Alternatively, MamE alone may be located in a vesicle that does not interact often with molecular motors and may need the presence of other magnetosome proteins such as MamI, MamL, or MamB, to localize to a vesicle that is more mobile. This may explain why MamE is not undergoing directed motion as often and why this fluorescent fusion protein is mostly immobile in the context of the reported trajectory analysis.

MamB particles undergoing active directed motion travel at 0.24 µm/s. From protein structure analysis [48,49], the proposed cytoplasmic domain of MamB is quite large and almost 20% of its amino acids have positive charges. These positive charges, therefore, have opportunities to interact with negatively-charged molecular motors in the mammalian intracellular compartment. We can thus infer that MamB either interacts directly with molecular motors or is located in a vesicle that interacts with molecular motors.

MamL particles undergoing active directed motion move at approximately 0.2 µm/s, and are thus expected to be interacting with molecular motors. Although the cationic amino acid residues of the MamB and MamL cytoplasmic domains are not arranged in a similar pattern [48,49], we can speculate that these two proteins may be interacting with similar molecular motors since they move at similar velocities. Interestingly, the directed motion of MamL_trunc_ particles (0.14 ± 0.06 µm/s) is not significantly different from the velocity of full-length MamL particles. Only the decrease in diffusion coefficient of MamL_trunc_ particles sets it apart from full-length protein (0.0019 ± 0.0011 vs. 0.0051 ± 0.0017 µm^2^/s; *p* < 0.001), implying a role of the cationic C-terminal peptide in regulating Brownian motion of MamL particles.

Tomato-MamL/EGFP-MamI and FLAG-MamL/EGFP-MamI particles undergoing active directed motion move at similar velocities as Tomato-MamL alone (0.23 µm/s vs. 0.14 µm/s vs. 0.20 µm/s, respectively). This suggests that Tomato-MamL alone, Tomato-MamL/EGFP-MamI, and FLAG-MamL/EGFP-MamI particles may all interact with the same molecular motors. Unlike the predicted protein structures of MamL, MamI has virtually no cytoplasmic domain [48,49], affording very little opportunity to interact with molecular motors [50]. Whether expressed alone or together with MamI, MamL is the protein most likely to interact with molecular motors. To this point, the interactions described for MamB and MamE with the mammalian intracellular compartment may serve as a baseline for comparison to the mobility of particles formed by the expression of multiple magnetosome proteins.

There are some exceptions and limitations to the general conclusions drawn from the above characterization of magnetosome proteins in a eukaryotic cell environment. When using ImageJ and Mathematica software to detect fluorescent particles, it was difficult to set parameters that included all visible trajectories. Instances where obvious trajectories were not detected included when the fluorescent particle was skipping frames and/or when the software confused it for another fluorescent particle travelling on a nearby trajectory. Whenever trajectories moved out of the focal plane (i.e., traveled along the z-axis) the fluorescence would dim and lead to potential loss of particle tracking and/or frame skipping of the timelapse video. The confocal microscope has great optical resolution in a narrow x-y focal plane; however, this limits our analysis to one narrow slice of the cell. Thus, the focal plane with the highest number of visible fluorescent particles was selected to represent the cell, and hundreds of trajectories per cell were analyzed. The microscope also has limitations to image resolution when acquiring timelapse videos in which the resolution must be lowered to preserve an appropriate frame rate (1 fps) for trajectory analysis. Finally, there is no data provided confirming protein interactions between mammalian molecular motors and any of the magnetosome proteins studied herein. Based on the trajectory analysis provided, there is every reason to expect that future research in this area will be valuable for understanding how essential magnetosome proteins interact in the eukaryotic cell.

In the future, the characterization of magnetosome particles could be further improved by employing more advanced methods for noise filtering, segmentation, linking and trajectory classification and analysis. In particular, the classification step could benefit from the use of machine learning approaches. In the present work, accurate trajectory classification required considering several observables (average and maximum step size, total displacement, anomalous exponent), but more systematic integration of multiple parameters (for example correlation in step direction or particle brightness) could improve both the precision and robustness of the classification. Machine learning approaches are well-suited for multi-dimensional analyses, and could help distinguish between diffusive, confined, and directed motion while reducing user bias [51,52].

## 5. Conclusions

The mobility of magnetosome proteins MamL, MamB, and MamE in a mammalian system indicates their possible interaction with mammalian molecular motors. The pattern of motion observed for the structures to which these proteins localize is consistent with the mobility of lipid vesicles in mammalian cells, namely a mixture of Brownian motion (with a very small diffusion coefficient on the order of 10^−3^ to 10^−2^ µm^2^/s) and directed motion (with a velocity on the order of 0.1 to 1 µm/s) [53,54]. Since MamL, MamB, and MamE all move at similar velocities when undergoing active directed motion, these magnetosome proteins may interact with the same type of molecular motor or localize to a structure that interacts with a common type of molecular motor. When co-expressed, MamI and MamL localize to the same intracellular compartment and form punctate, mobile structures that move at the same velocity as particles expressing MamL alone, again suggesting interactions with a common molecular motor. Taken together, this trajectory analysis supports mounting evidence that magnetosome proteins are compatible with mammalian cell systems and localize within their membranous compartments.

## Figures and Tables

**Table 1:** Primer design for cloning MTB genes *mamB* and *mamE*.

Gene	Primer (5′–3′)		Restriction Site *	Vector
mamB	Forward	CAATCTTGTG**GAATTC**AGAACCG	EcoRI	ptdTomato-C1
	Reverse	ACGCTCTGG**CCCGGG**ATGTCC	SmaI	
mamE	Forward	ACCCTG**AGATCT**GGATGGTTG	BglII	pEGFP-C1
	Reverse	GCCATTATCC**GAGCTC**CACCA	SacI	

* Restriction enzyme sites appear in bold within the primer sequence.

**Table 2:** Summary of trajectory analysis in mammalian cells expressing magnetosome proteins.

Fusion Protein	Number of Cells	* Number of Analyzed Trajectories	Percent Immobile	Percent Brownian	Percent Directed	** Apparent Diffusion Coefficient (10^−3^ µm^2^/s)	** α Value for the Apparent Diffusion Coefficient	*** Velocity (µm/s)
EGFP-MamE	4	47	26	62	13	1.9 ± 0.4 ^†,§^	0.15 ± 0.04	0.17 ± 0.03
Tomato-MamB	5	571	15	57	28	4.0 ± 1.0 ^§^	0.54 ± 0.08	0.24 ± 0.01
Tomato-MamL	7	461	18	56	25	5.1 ± 1.7 ^†,‡^	0.43 ± 0.17	0.19 ± 0.05
Tomato-MamL_trunc_	7	516	14	60	26	1.9 ± 1.1 ^‡^	0.52 ± 0.11	0.14 ± 0.06
Tomato-MamL + EGFP-MamI	4	225	21	45	32	3.2 ± 2.5	0.42 ± 0.11	0.23 ± 0.09
FLAG-MamL + EGFP-MamI	5	520	13	66	21	5.0 ± 0.9	0.41 ± 0.11	0.14 ± 0.05

* The total number of analyzed trajectories is calculated in Mathematica. ** Apparent diffusion coefficient and anomalous exponent (α value) are measured from particles undergoing Brownian motion. Data are the mean ± standard deviation. *** Velocity is measured from particles undergoing directed motion. Data are the mean ± standard deviation. ^†,‡^ *p* < 0.001. ^§^ *p* < 0.05.

**Table 3:** Summary of trajectory analysis in mammalian cells after colchicine treatment.

	Number of Cells Analyzed	* Number of Trajectories Analyzed	% Immobile	% Brownian	% Directed	** Apparent Diffusion Coefficient (10^−3^ µm^2^/s)	*** Maximum Velocity (µm/s)
**** MamL	7	461	18	56	26	5.1 ± 1.7	0.19 ± 0.05
**MamL + ** **colchicine**	6	297	30	65	5	4.3 ± 0.8	0.11 ± 0.04
**** MamL + I	5	120	0	22	78	4.6 ± 2.4 ^§^	0.14 ± 0.03 ^†^
**MamL + I ** **+ colchicine**	5	64	19	50	31	1.8 ± 0.4 ^§^	0.05 ± 0.02 ^†^

* The total number of analyzed trajectories is calculated in Mathematica. ** Apparent diffusion coefficient is measured from particles undergoing Brownian motion. Data are the mean ± standard deviation. *** Velocity is measured from particles undergoing directed motion. Data are the mean ± standard deviation. **** Data for MamL and MamL + I (no colchicine) were previously presented in Table 2. ^§^ *p* < 0.05. ^†^ *p* < 0.001.

## Data Availability

The sequence of each construct can be accessed in the Electronic Thesis and Dissertation Repository maintained by Scholarship@Western using the following URL: https://hdl.handle.net/20.500.14721/32958 (accessed on 5 April 2023) (Sun, 2023). The Mathematica code for trajectory analysis can be accessed at the following link: https://github.com/cecilefradin/Particle-Trajectory-Classification-and-Analysis (accessed on 5 April 2023).

## Supplementary Materials

The following supporting information can be downloaded at: https://www.mdpi.com/article/10.3390/bios15120797/s1, Figure S1: MamE sequence alignment and homology across several magnetotactic bacterial species; Figure S2: ImageJ Mosaic Particle Tracker user interface; Figure S3: Immunoblot of total cellular protein from mammalian cells expressing EGFP-MamE; Figure S4: Immunoblot of total cellular protein from mammalian cells expressing Tomato-MamB; Figure S5: Trajectory analysis of EGFP-MamE particles; Figure S6: Trajectory analysis of Tomato-MamB particles; Figure S7: Trajectory analysis of Tomato-MamL particles; Figure S8: Trajectory analysis of Tomato-MamL_trunc_ particles; Figure S9: Trajectory analysis of Tomato-MamL/EGFP-MamI particles; Figure S10: Trajectory analysis of FLAG-MamL/EGFP-MamI particles; Figure S11: Trajectory Analysis of Tomato-MamL and FLAG-MamL+EGFP-MamI particles before and after colchicine treatment; Figure S12: Trajectory Analysis of Tomato-MamL particles before and after colchicine treatment; Figure S13: Trajectory Analysis of FLAG-MamL and MamI particles before and after colchicine treatment; Figure S14: Comparison of trajectory parameters for individual particles detected in cells expressing magnetosome proteins; Table S1: Trajectory analysis of mammalian cells expressing magnetosome proteins that move along microtubules; Table S2: Summary of velocity and anomalous exponent values for particles undergoing directed motion; Video S1: MamE/10X speed; Video S2: MamB/10X speed; Video S3: MamL microtubules/10X speed; Video S4: MamIL microtubules/10X speed.
