# Supplementary material for: Cellular Distribution and Motion of Essential Magnetosome Proteins Expressed in Mammalian Cells
Source: Biosensors (Basel). 2025 Dec 4;15(12):797. doi: 10.3390/bios15120797 (PMC12730242; doi:10.3390/bios15120797)
Supplement: Supplementary file 1 [file biosensors-15-00797-s001.zip › biosensors-3934541-supplementary.pdf]

Supplementary Information

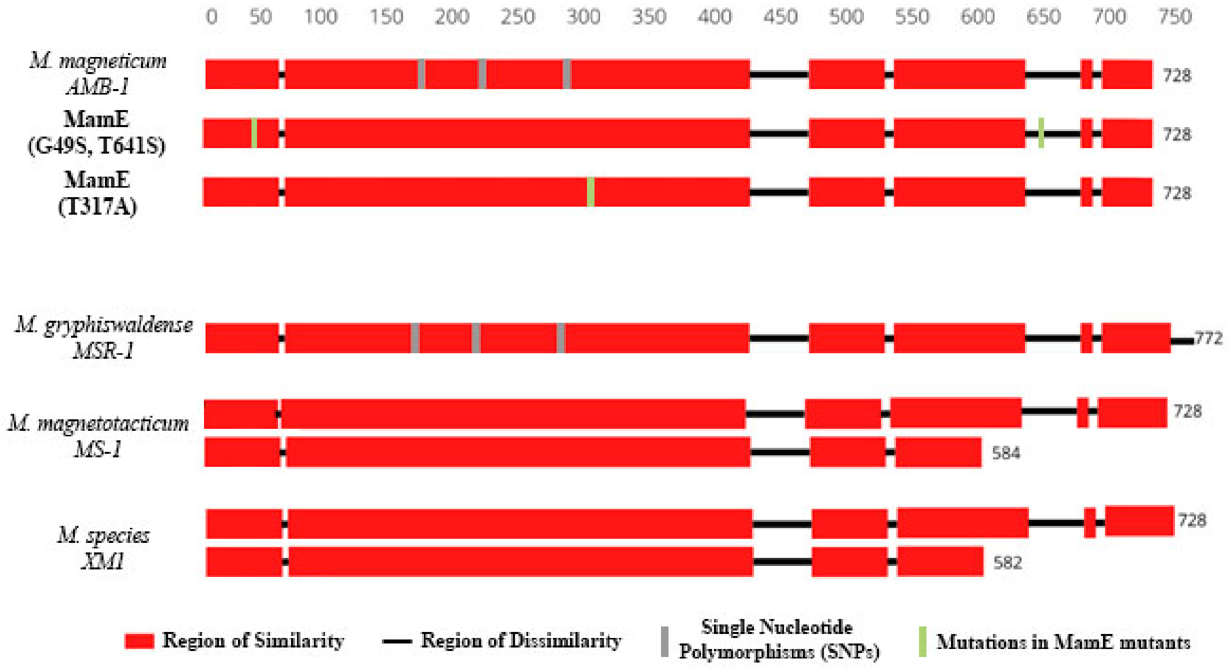

Supplementary Figure S1. MamE sequence alignment and homology across several magnetotactic bacterial species.
Regions of similarity in the amino acid sequence of MamE across species are highlighted in red, while regions of dissimilarity are in black, and single nucleotide polymorphisms (SNPs) appear in grey. Minor changes in the sequence of MamE (G49S, T641S) and MamE (T317A) are indicated in green and compared to the sequence from *Magnetospirillum magneticum* AMB-1 from which they were cloned. While the conservative substitution (T641S) in a region of variability is not expected to influence MamE function, neither substitution in different conserved regions (G49S or T317A) altered the trajectory analysis of MamE expressed in mammalian cells.

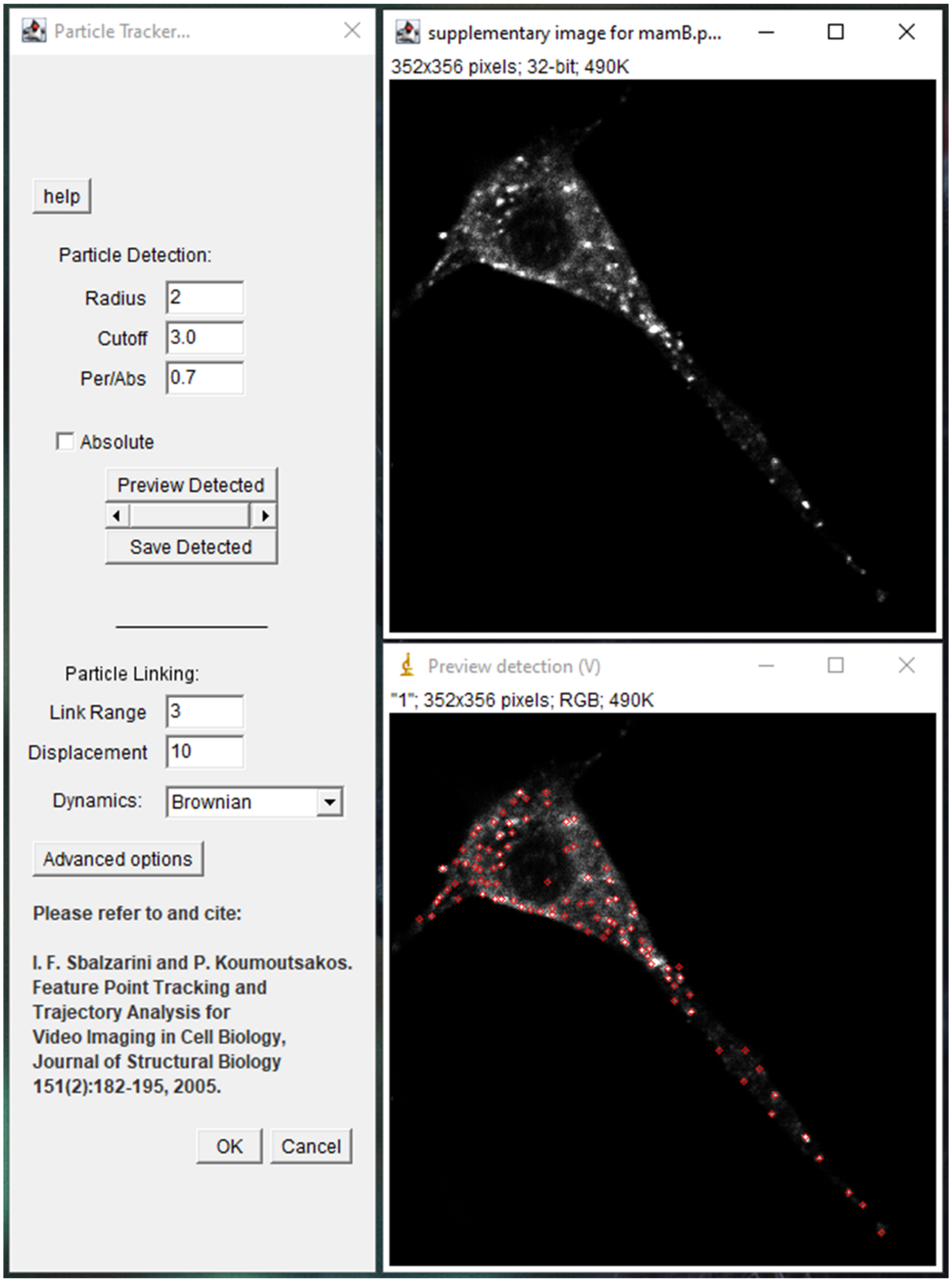

Supplementary Figure S2. ImageJ Mosaic Particle Tracker user interface. A representative frame is shown from a movie of MDA-MB-435 cells expressing Tomato-MamB. The movie is opened in the software and converted to greyscale (top right panel). After setting the detection parameters (left panel), the software delineates punctate structures with red circles (bottom right panel) and initiates the trajectory analysis.

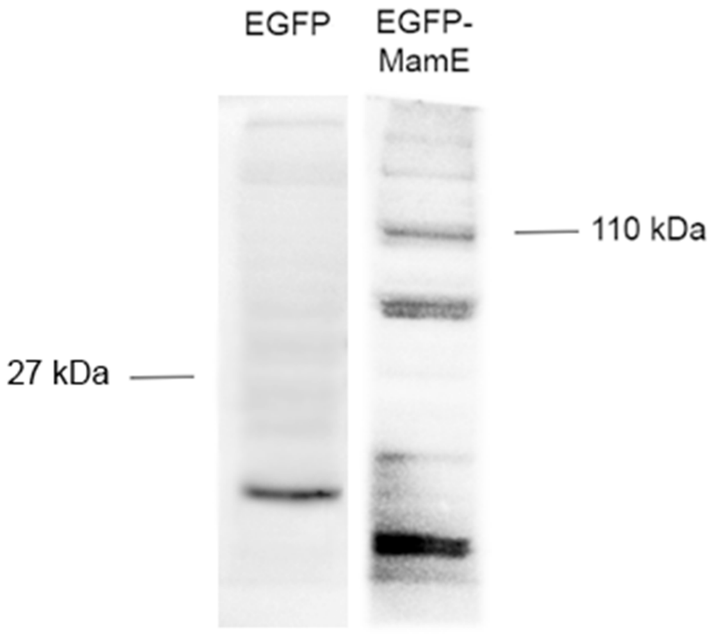

Supplementary Figure S3. Immunoblot of total cellular protein from mammalian cells expressing EGFP-MamE. Total cellular protein from MDA-MB-435 cells stably expressing EGFP-MamE was examined with mouse α-EGFP. The full-length western blot is displayed.

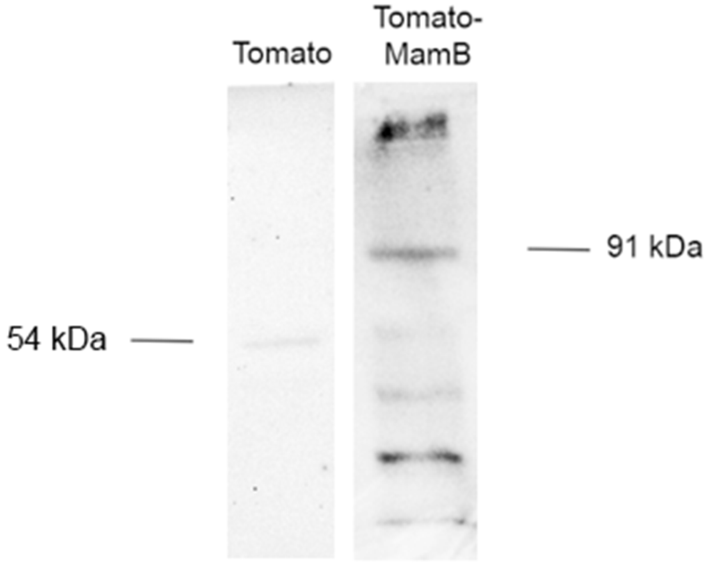

Supplementary Figure S4. Immunoblot of total cellular protein from mammalian cells expressing Tomato-MamB. Total cellular protein from MDA-MB-435 cells stably expressing Tomato-MamB was examined with rabbit α-Tomato. The full-length western blot is displayed.

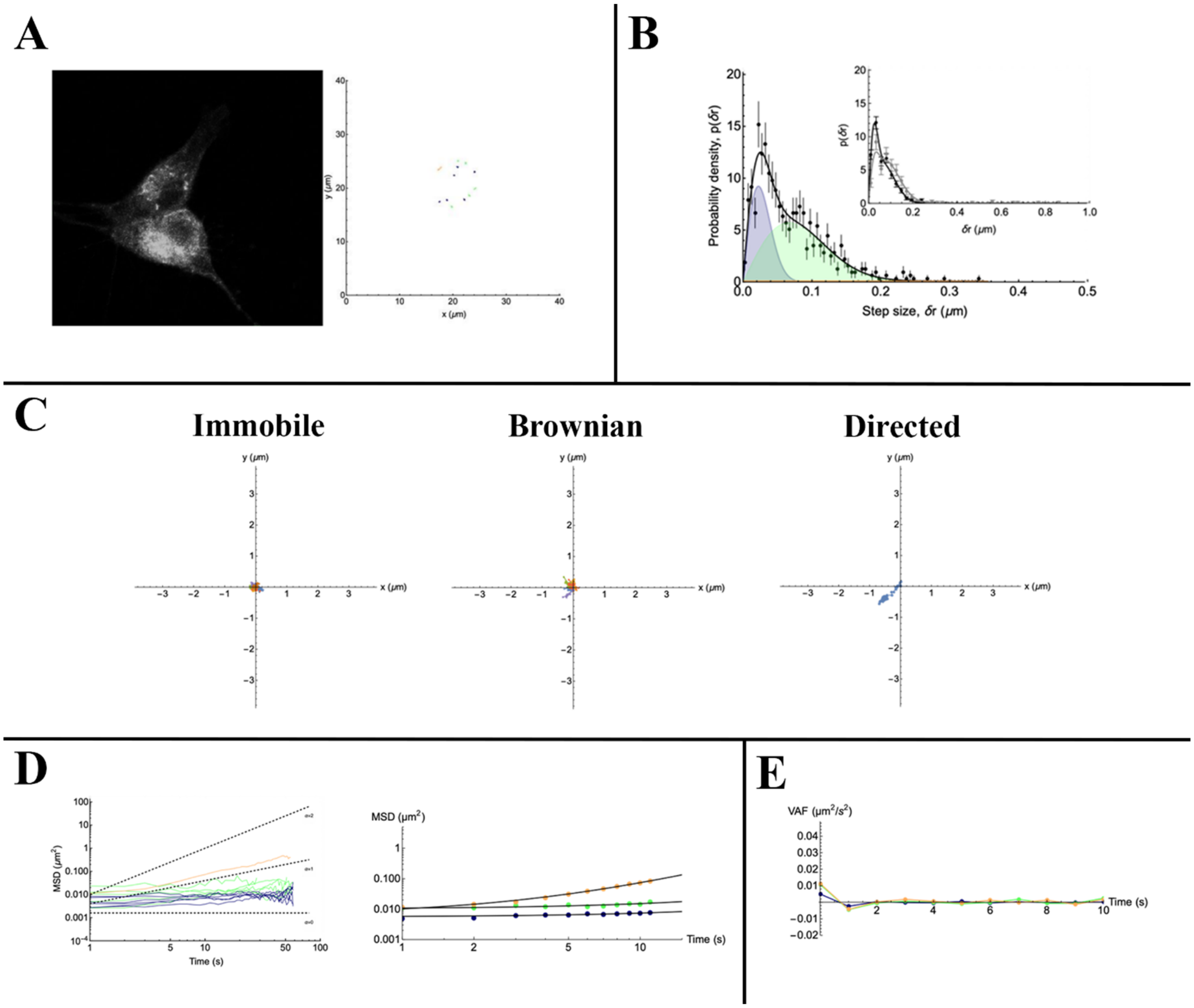

Supplementary Figure S5: Trajectory analysis of EGFP-MamE particles.
A. The confocal micrograph of a representative cell (left panel, black and white image of Figure 3A) is shown with trajectories of punctate particles detected within the cell (right panel). Immobile, Brownian, and directed trajectories are represented in black, green, and orange, respectively. **B.** The representative distribution of displacements is presented, where the immobile, Brownian, and direct populations are represented by the blue, green, and orange peaks, respectively. **C.** Immobile, Brownian, and directed trajectories are plotted on a cartesian plane. **D.** Immobile (black), Brownian (green), and direct (orange) trajectories are represented as mean-squared displacement (MSD) as a function of lag time. Dashed lines indicate the expected slope of the MSD, and the figure on the right shows average MSD for all trajectories. **E.** The plot shows average velocity autocorrelation function (VAF) of immobile (black), Brownian (green), and direct (orange) trajectories.

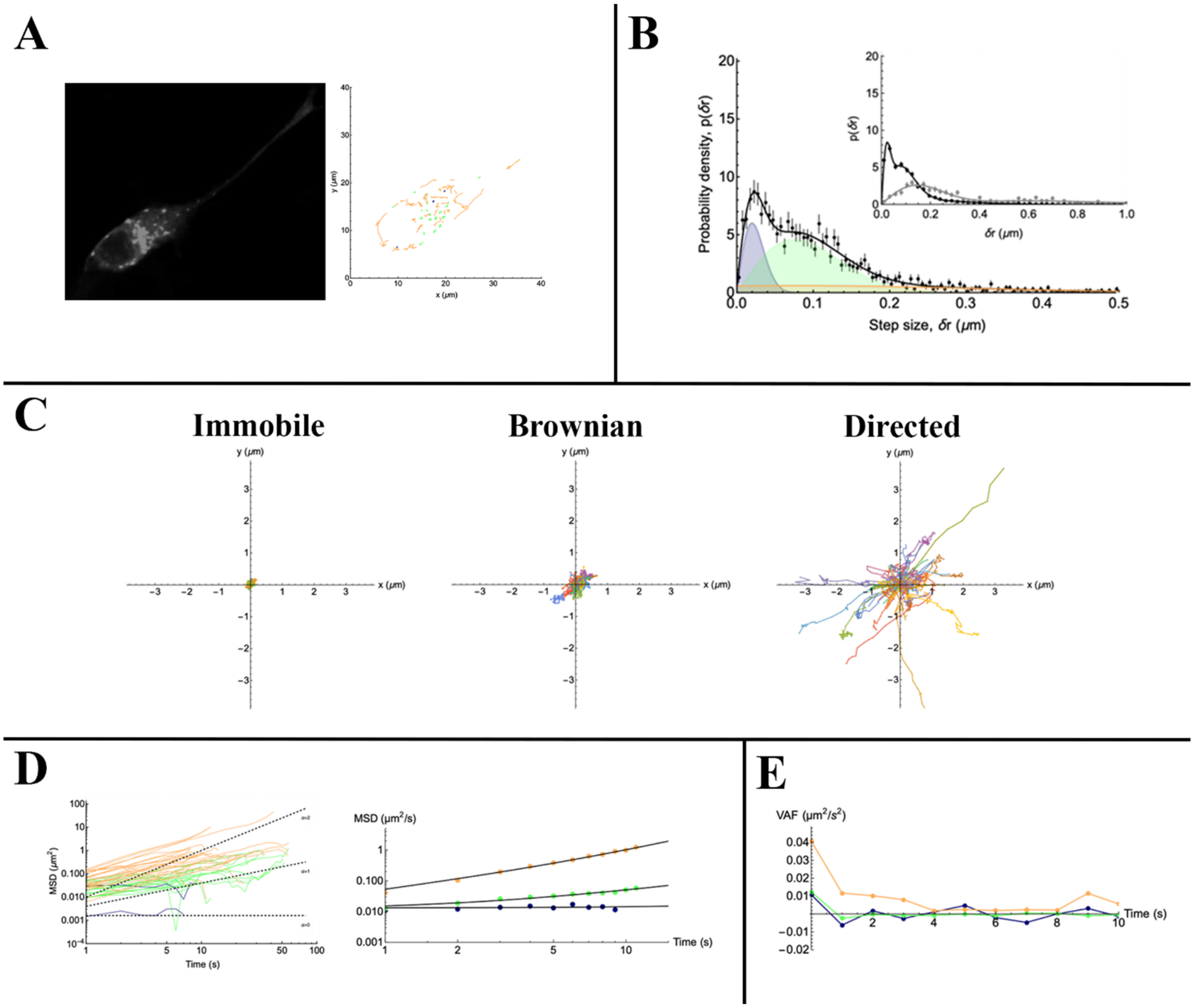

Supplementary Figure S6: Trajectory analysis of Tomato-MamB particles.
A. The confocal micrograph of a representative cell (left panel, black and white image of Figure 4A) is shown with trajectories of punctate particles detected within the cell (right panel). Immobile, Brownian, and directed trajectories are represented in black, green, and orange, respectively. **B.** The representative distribution of displacements is presented, where the immobile, Brownian, and direct populations are represented by the blue, green, and orange peaks, respectively. **C.** Immobile, Brownian, and directed trajectories are plotted on a cartesian plane. **D.** Immobile (black), Brownian (green), and direct (orange) trajectories are represented as mean-squared displacement (MSD) as a function of lag time. Dashed lines indicate the expected slope of the MSD, and the figure on the right shows average MSD for all trajectories. **E.** The plot shows average velocity autocorrelation function (VAF) of immobile (black), Brownian (green), and direct (orange) trajectories.

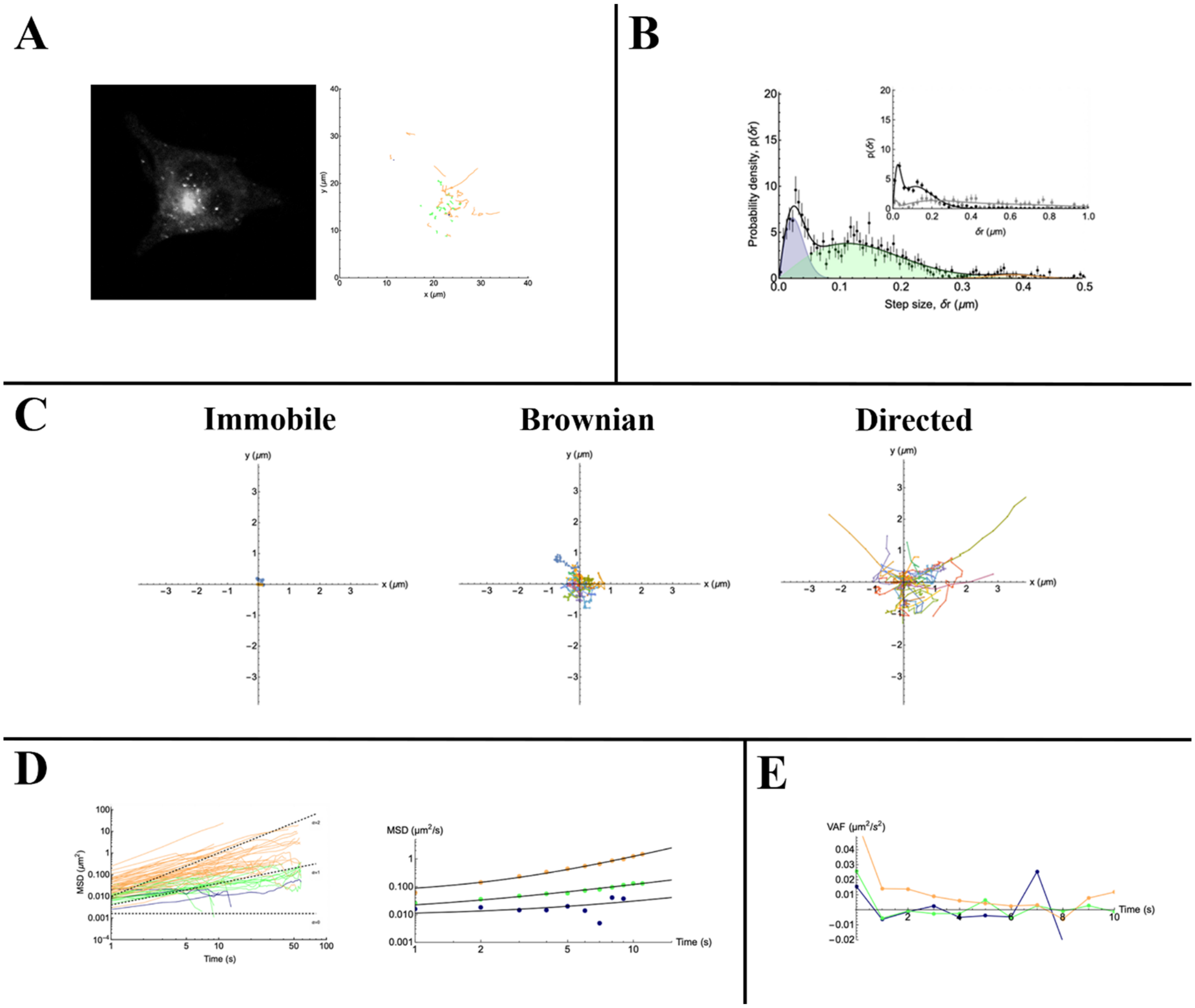

Supplementary Figure S7: Trajectory analysis of Tomato-MamL particles.
A. The confocal micrograph of a representative cell (left panel, black and white image of Figure 5A) is shown with trajectories of punctate particles detected within the cell (right panel). Immobile, Brownian, and directed trajectories are represented in black, green, and orange, respectively. **B.** The representative distribution of displacements is presented, where the immobile, Brownian, and direct populations are represented by the blue, green, and orange peaks, respectively. **C.** Immobile, Brownian, and directed trajectories are plotted on a cartesian plane. **D.** Immobile (black), Brownian (green), and direct (orange) trajectories are represented as mean-squared displacement (MSD) as a function of lag time. Dashed lines indicate the expected slope of the MSD, and the figure on the right shows average MSD for all trajectories. **E.** The plot shows average velocity autocorrelation function (VAF) of immobile (black), Brownian (green), and direct (orange) trajectories.

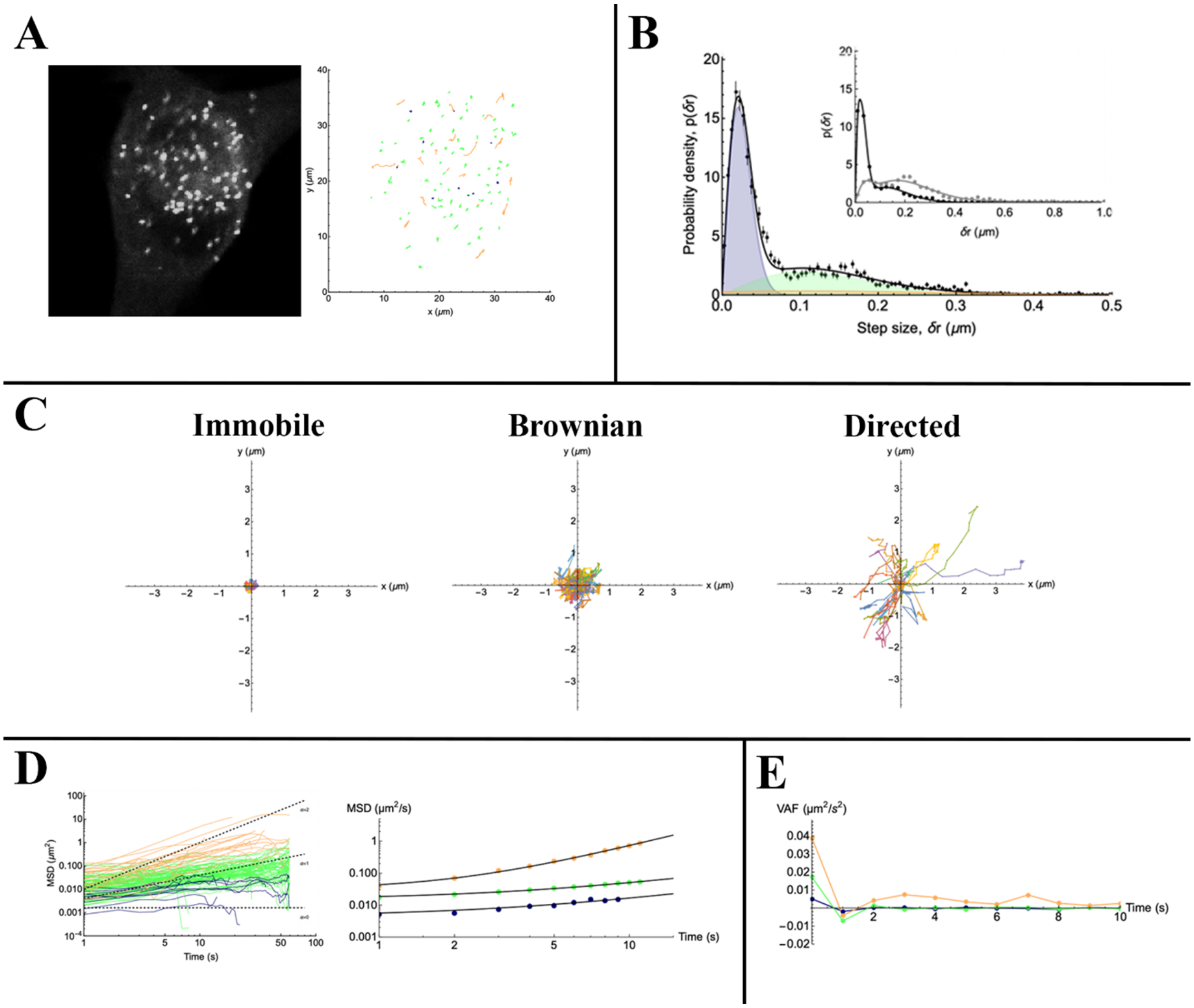

Supplementary Figure S8: Trajectory analysis of Tomato-MamL$_{trunc}$ particles.
A. The confocal micrograph of a representative cell (left panel, black and white image of Figure 5D) is shown with trajectories of punctate particles detected within the cell (right panel). Immobile, Brownian, and directed trajectories are represented in black, green, and orange, respectively. **B.** The representative distribution of displacements is presented, where the immobile, Brownian, and direct populations are represented by the blue, green, and orange peaks, respectively. **C.** Immobile, Brownian, and directed trajectories are plotted on a cartesian plane. **D.** Immobile (black), Brownian (green), and direct (orange) trajectories are represented as mean-squared displacement (MSD) as a function of lag time. Dashed lines indicate the expected slope of the MSD, and the figure on the right shows average MSD for all trajectories. **E.** The plot shows average velocity autocorrelation function (VAF) of immobile (black), Brownian (green), and direct (orange) trajectories.

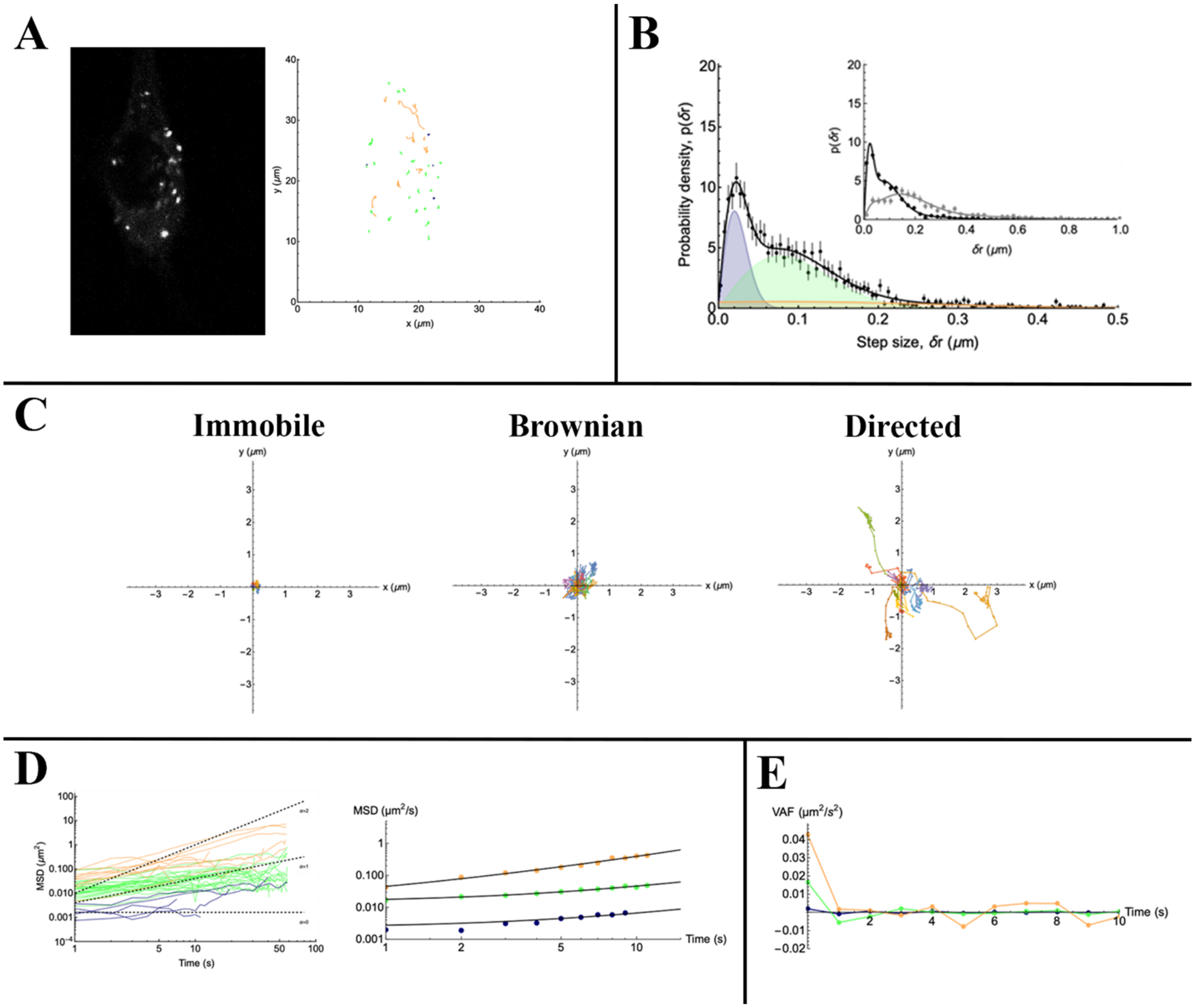

Supplementary Figure S9: Trajectory analysis of Tomato-MamL/EGFP-MamI particles. **A.** The confocal micrograph of a representative cell (left panel, black and white image of Figure 6A) is shown with trajectories of punctate particles detected within the cell (right panel). Immobile, Brownian, and directed trajectories are represented in black, green, and orange, respectively. **B.** The representative distribution of displacements is presented, where the immobile, Brownian, and direct populations are represented by the blue, green, and orange peaks, respectively. **C.** Immobile, Brownian, and directed trajectories are plotted on a cartesian plane. **D.** Immobile (black), Brownian (green), and direct (orange) trajectories are represented as mean-squared displacement (MSD) as a function of lag time. Dashed lines indicate the expected slope of the MSD, and the figure on the right shows average MSD for all trajectories. **E.** The plot shows average velocity autocorrelation function (VAF) of immobile (black), Brownian (green), and direct (orange) trajectories.

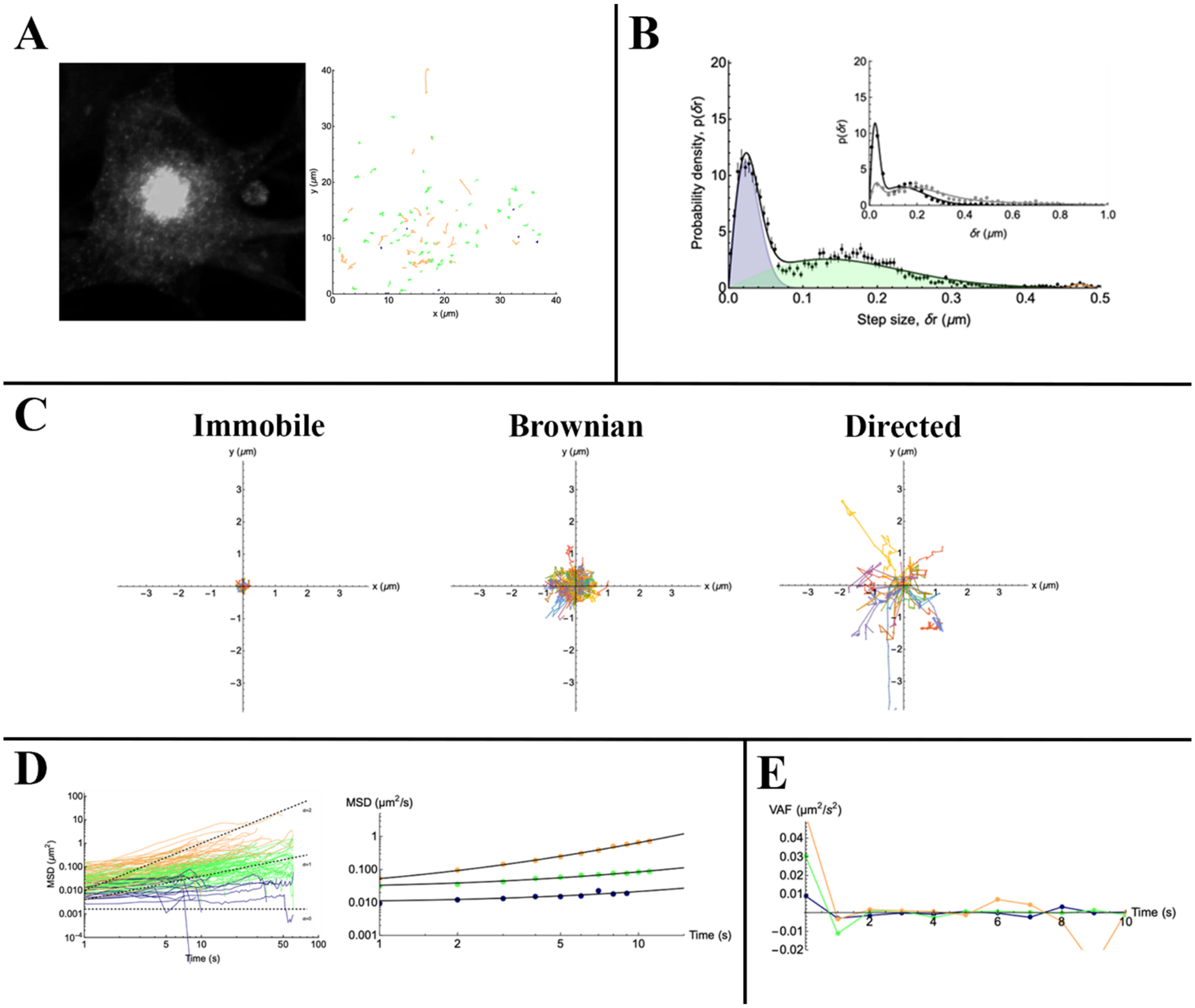

Supplementary Figure S10: Trajectory analysis of FLAG-MamL/EGFP-MamI particles. **A.** The confocal micrograph of a representative cell (left panel, black and white image of Figure 6D) is shown with trajectories of punctate particles detected within the cell (right panel). Immobile, Brownian, and directed trajectories are represented in black, green, and orange, respectively. **B.** The representative distribution of displacements is presented, where the immobile, Brownian, and direct populations are represented by the blue, green, and orange peaks, respectively. **C.** Immobile, Brownian, and directed trajectories are plotted on a cartesian plane. **D.** Immobile (black), Brownian (green), and direct (orange) trajectories are represented as mean-squared displacement (MSD) as a function of lag time. Dashed lines indicate the expected slope of the MSD, and the figure on the right shows average MSD for all trajectories. **E.** The plot shows average velocity autocorrelation function (VAF) of immobile (black), Brownian (green), and direct (orange) trajectories.

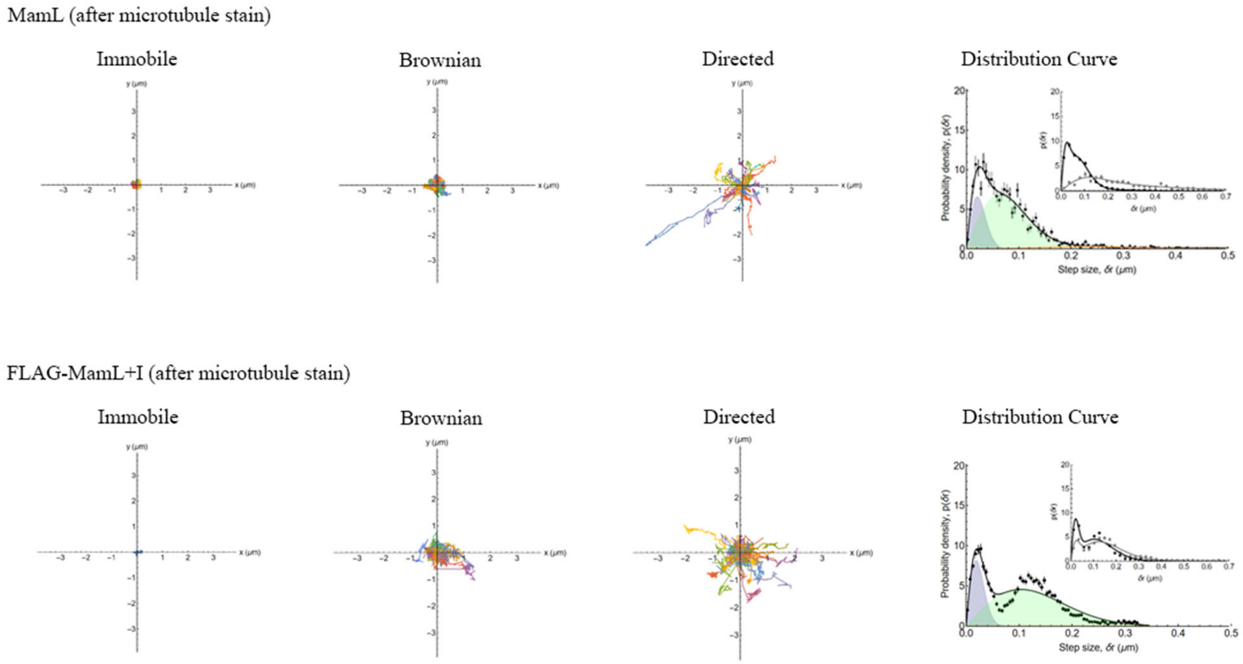

Supplementary Figure S11. Trajectory Analysis of Tomato-MamL and FLAG-MamL+EGFP-MamI particles before and after colchicine treatment. Immobile, Brownian, and directed trajectories are plotted on a cartesian plane. The representative distribution of displacement is presented on the right, where the immobile, Brownian, and direct populations are represented by the blue, green, and orange peaks, respectively. There is a decrease in directed and Brownian trajectories after colchicine treatment, and an increase in immobile particles.

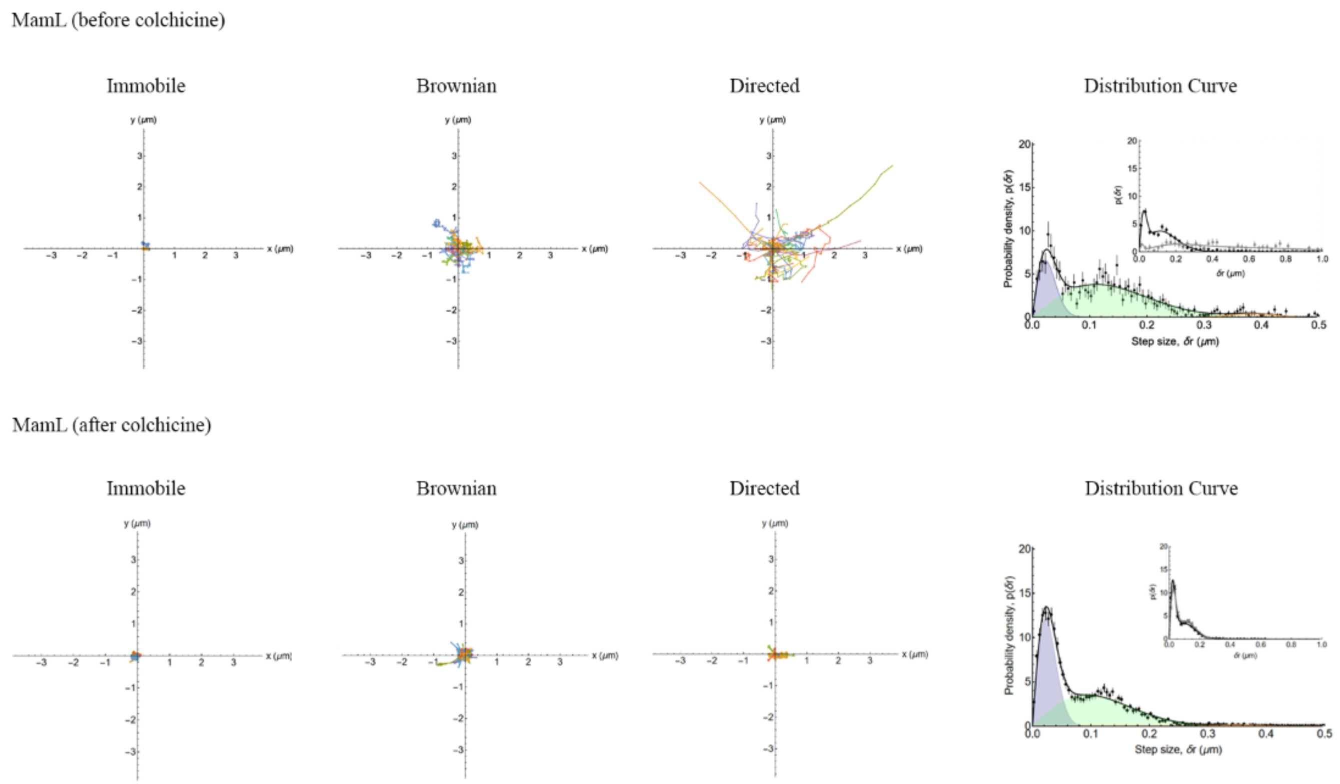

Supplementary Figure S12. Trajectory Analysis of Tomato-MamL particles before and after colchicine treatment. Immobile, Brownian, and directed trajectories are plotted on a cartesian plane. The representative distribution of displacement is presented on the right, where the immobile, Brownian, and direct populations are represented by the blue, green, and orange peaks, respectively. There is a decrease in directed and Brownian trajectories after colchicine treatment, and an increase in immobile particles.

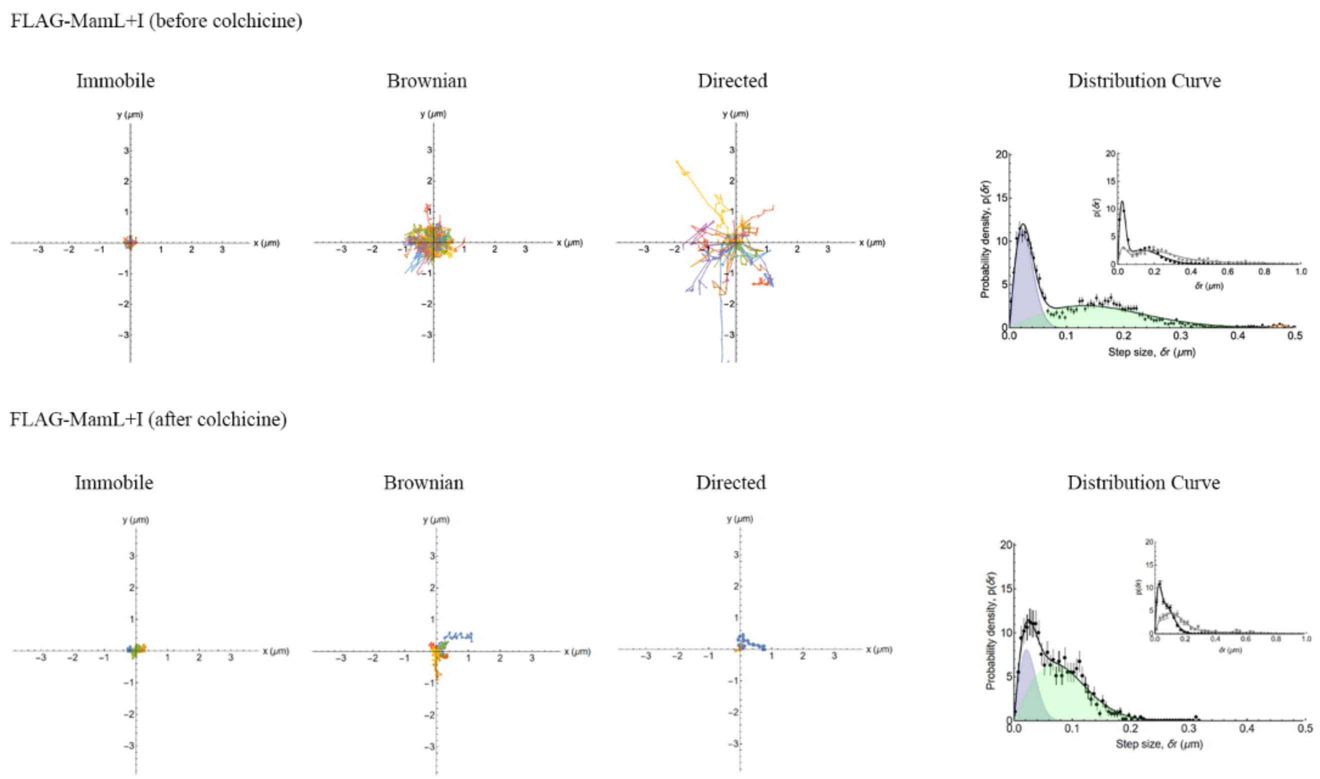

Supplementary Figure S13. Trajectory Analysis of FLAG-MamL and MamI particles before and after colchicine treatment. Immobile, Brownian, and directed trajectories are plotted on a cartesian plane. The representative distribution of displacement is presented on the right, where the immobile, Brownian, and direct populations are represented by the blue, green, and orange peaks, respectively. There is a decrease in directed and Brownian trajectories after colchicine treatment, and an increase in immobile particles.

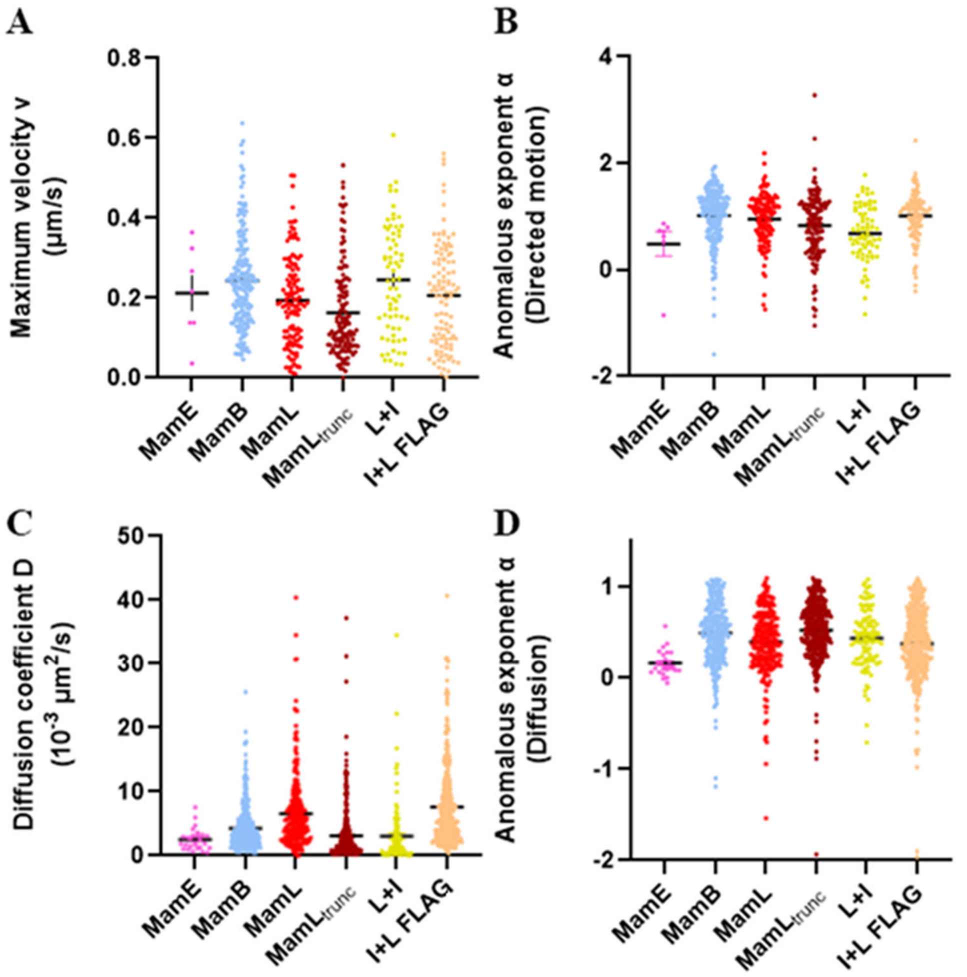

Supplementary Figure S14: Comparison of trajectory parameters for individual particles detected in cells expressing magnetosome proteins. In trajectories classified as directed motion, scatter plots indicate the range in both maximum velocity (A) and anomalous exponent (B). Likewise, in trajectories classified as entirely Brownian, the range in both diffusion coefficients (C) and anomalous exponents (D) were extracted.

Table S1. Trajectory analysis of mammalian cells expressing magnetosome proteins that move along microtubules.

Fusion Protein	Number of cells	* Number of analyzed trajectories	Percent immobile	Percent Brownian	Percent directed	**Apparent diffusion coefficient (10^{-3} μm^2/s)	**α Value for the apparent diffusion coefficient	***Velocity (μm/s)
**Tomato-MamL**	6	502	5	36	59	5.4 ± 3.3	0.46 ± 0.16	0.16 ± 0.05
**FLAG-MamL + EGFP-MamI**	7	983	1	34	65	9.9 ± 4.5	0.35 ± 0.18	0.24 ± 0.07

* Prior to trajectory analysis, cells were stained with ViaFluor 647 Live Cell Microtubule Stain.

** Apparent diffusion coefficient and anomalous exponent (α value) are measured from particles undergoing Brownian motion. Data are the mean ± standard deviation.

*** Velocity is measured from particles undergoing directed motion. Data are the mean ± standard deviation.

Table S2. Summary of velocity and anomalous exponent values for particles undergoing directed motion.

Magnetosome protein	% Particles undergoing directed motion	* Velocity (µm/s)	* α Value
**EGFP-MamE**	13	0.17 ± 0.03	0.26 ± 0.32
**Tomato-MamB**	28	0.24 ± 0.01	1.24 ± 0.04
**Tomato-MamL**	25	0.19 ± 0.05	1.01 ± 0.18
**Tomato-MamL$_{trunc}$**	26	0.14 ± 0.06	0.88 ± 0.31
**Tomato-MamL + EGFP-MamI**	32	0.23 ± 0.09	0.66 ± 0.10
**FLAG-MamL + EGFP-MamI**	21	0.14 ± 0.05	0.95 ± 0.33

* Velocity and anomalous exponent (α value) are measured from particles undergoing directed motion. Data are the mean ± standard deviation.
