# Supplementary figures and images for: Cellular Distribution and Motion of Essential Magnetosome Proteins Expressed in Mammalian Cells
Source: Biosensors (Basel). 2025 Dec 4;15(12):797. doi: 10.3390/bios15120797 (PMC12730242; doi:10.3390/bios15120797)

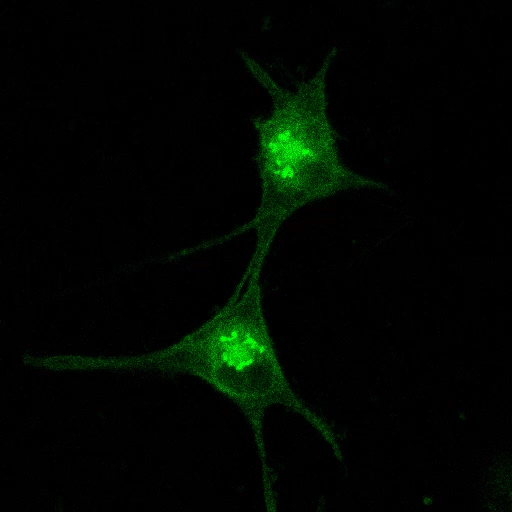

Supplement: Supplementary file 1 [file biosensors-15-00797-s001.zip › Video-S1-MamE 10X speed.gif]

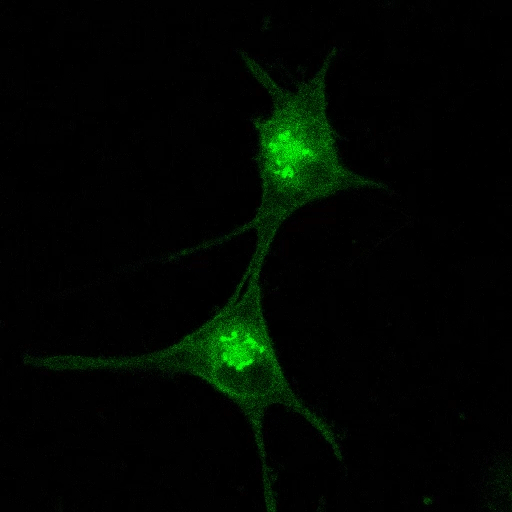

Supplement: Supplementary file 1 [file biosensors-15-00797-s001.zip › Video-S1-MamE.gif]

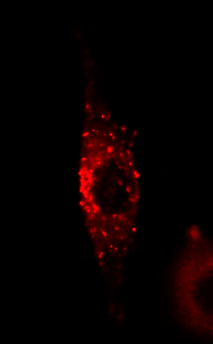

Supplement: Supplementary file 1 [file biosensors-15-00797-s001.zip › Video-S2-MamB 10X speed.gif]

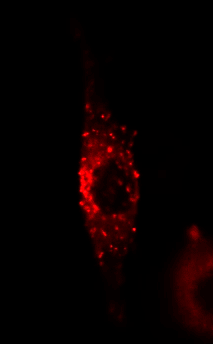

Supplement: Supplementary file 1 [file biosensors-15-00797-s001.zip › Video-S2-MamB.gif]

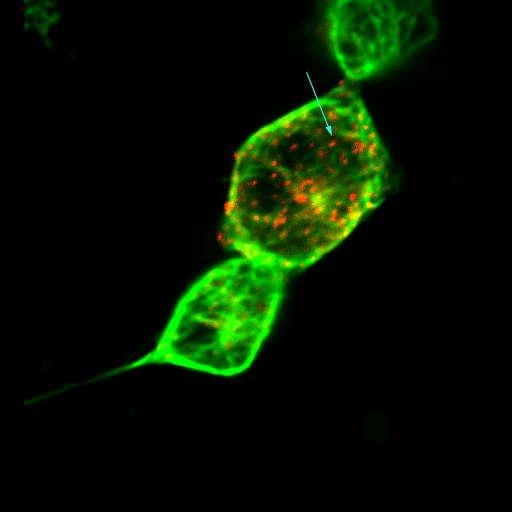

Supplement: Supplementary file 1 [file biosensors-15-00797-s001.zip › Video-S3-MamL microtubules 10X speed.gif]

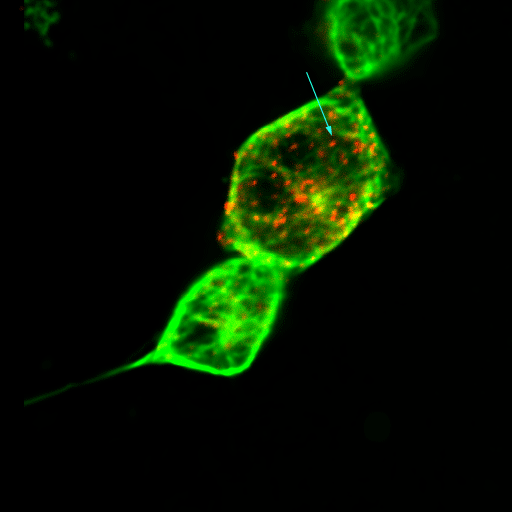

Supplement: Supplementary file 1 [file biosensors-15-00797-s001.zip › Video-S3-MamL microtubules.gif]

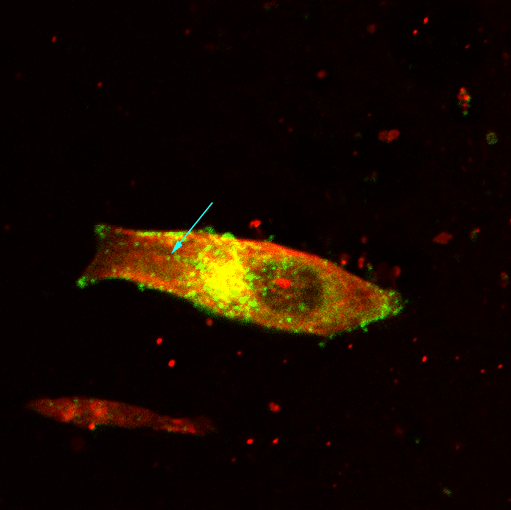

Supplement: Supplementary file 1 [file biosensors-15-00797-s001.zip › Video-S4-MamIL microtubules 10X speed.gif]

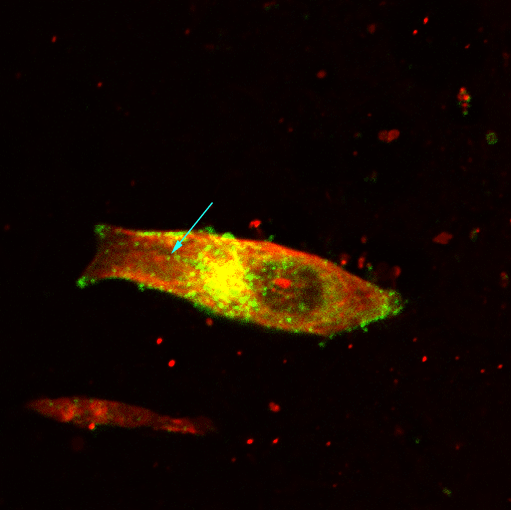

Supplement: Supplementary file 1 [file biosensors-15-00797-s001.zip › Video-S4-MamIL microtubules.gif]
